# Targeting positive feedback between BASP1 and EGFR as a therapeutic strategy for lung cancer progression

**DOI:** 10.7150/thno.49425

**Published:** 2020-08-29

**Authors:** Ching-Chan Lin, Yu-Kai Huang, Chia-Fong Cho, Yu-Sen Lin, Chia-Chien Lo, Ting-Ting Kuo, Guan-Chin Tseng, Wei-Chung Cheng, Wei-Chao Chang, Tzu-Hung Hsiao, Liang-Chuan Lai, Jin-Yuan Shih, Yu-Huei Liu, K.S. Clifford Chao, Jennifer L. Hsu, Pei-Chih Lee, Xian Sun, Mien-Chie Hung, Yuh-Pyng Sher

**Affiliations:** 1 Graduate Institute of Clinical Medical Science, China Medical University, Taichung 404, Taiwan.; 2 Graduate Institute of Biomedical Sciences, China Medical University, Taichung 404, Taiwan.; 3 Chinese Medicine Research Center, China Medical University, Taichung 404, Taiwan.; 4 Research Center for Chinese Herbal Medicine, China Medical University, Taichung 404, Taiwan.; 5 Graduate Institute of Integrated Medicine, China Medical University, Taichung 404, Taiwan.; 6 Research Center for Tumor Medical Science, China Medical University, Taichung 404, Taiwan.; 7 Drug Development Center, China Medical University, Taichung 404, Taiwan.; 8 Division of Hematology and Oncology, China Medical University Hospital, Taichung 404, Taiwan.; 9 Division of Thoracic Surgery, China Medical University Hospital, Taichung 404, Taiwan.; 10 Center for Molecular Medicine, China Medical University Hospital, Taichung 404, Taiwan.; 11 Department of Anatomic Pathology, Nantou Hospital of the Ministry of Health and Welfare, Nantou 540, Taiwan.; 12 Department of Medical Genetics and Medical Research, China Medical University Hospital, Taichung 404, Taiwan.; 13 Cancer Center, China Medical University Hospital, Taichung 404, Taiwan.; 14 Department of Medical Research, Taichung Veterans General Hospital, Taichung 407, Taiwan.; 15 Graduate Institute of Physiology, College of Medicine, National Taiwan University, Taipei 100, Taiwan.; 16 Graduate Institute of Clinical Medicine, National Taiwan University, Taipei 106, Taiwan.; 17 Department of Internal Medicine, National Taiwan University Hospital, Taipei 106, Taiwan.; 18 Department of Molecular and Cellular Oncology, The University of Texas MD Anderson Cancer Center, Houston, Texas 77030, USA.; 19 Department of Medical Oncology, Harbin Medical University Cancer Hospital, Harbin, China.

**Keywords:** lung adenocarcinoma, BASP1, arsenic trioxide, EGFR-TKI acquired resistance, combination therapy

## Abstract

**Rationale:** Brain metastasis in patients with lung cancer is life-threatening. However, the molecular mechanism for this catastrophic disease remains elusive, and few druggable targets are available. Therefore, this study aimed to identify and characterize proteins that could be used as therapeutic targets.

**Methods:** Proteomic analyses were conducted to identify differentially expressed membrane proteins between brain metastatic lung cancer cells and primary lung cancer cells. A neuronal growth-associated protein, brain acid soluble protein 1 (BASP1), was chosen for further investigation. The clinical relevance of BASP1 in lung adenocarcinoma was first assessed. Tyrosine kinase activity assays and *in vitro* and *in vivo* functional assays were conducted to explore the oncogenic mechanisms of BASP1.

**Results:** The protein levels of BASP1 were positively associated with tumor progression and poor prognosis in patients with lung adenocarcinoma. Membrane-bound BASP1 increased EGFR signaling and stabilized EGFR proteins by facilitating their escape from the ubiquitin-proteasome pathway. Reciprocally, activation of EGFR recruited more BASP1 to the plasma membrane, generating a positive feedback loop between BASP1 and EGFR. Moreover, the synergistic therapeutic effects of EGFR tyrosine kinase inhibitor and arsenic trioxide led to a reduction in the level of BASP1 protein observed in lung cancer cells with acquired resistance to EGFR inhibitors.

**Conclusions:** The reciprocal interaction between BASP1 and EGFR facilitates EGFR signaling in brain metastatic lung cancer. Targeting the newly identified BASP1-EGFR interaction could open new venues for lung cancer treatment.

## Introduction

Metastatic lung cancer remains the deadliest cancer in the world, with a five-year survival rate of only 5% 1. Non-small-cell lung cancer (NSCLC) accounts for approximately 70-80% of all lung cancers 2 and oncogenic activation of receptor tyrosine kinases (RTKs), such as epidermal growth factor receptor (EGFR), is especially relevant for this disease 3. Specifically, overexpression and/or activating mutations of EGFR occur in approximately 30-60% of East Asian and 8-15% of Caucasian patients with advanced NSCLC 1, 4. Tyrosine kinase inhibitors (TKIs) targeting EGFR were used as the first-line treatment for patients with metastatic NSCLC whose tumors harbor EGFR mutations 5; however, those patients eventually develop acquired resistance through either secondary EGFR mutations, e.g., T790M, or activation of the bypass track signaling pathways, such as activation of RTK AXL to maintain persistent oncogenic EGFR signaling 6. Overcoming alternative survival signaling pathways for activating the EGFR signaling network in lung cancer progression may lead to more effective therapeutic strategies.

Brain acid soluble protein-1 (BASP1) belongs to the family of neuronal growth-associated proteins, which also includes myristoylated alanine rich protein kinase C substrate (MARCKS) and growth-associated protein 43 (GAP43). These proteins share remarkably similar roles in actin regulation, neurite outgrowth, and anatomical plasticity in neural cells 7. Although BASP1, MARCKS, and GAP43 are also expressed in non-nerve tissues, their functions in cancers are distinct. In human tumors, the properties of GAP43 and MARCKS are primarily oncogenic 8. In contrast, nuclear BASP1 inhibits Myc-induced fibroblast transformation 9, suppresses the proliferation of acute myeloid leukemia 10, and acts as a transcriptional corepressor in breast cancer 11, suggesting that BASP1 harbors tumor inhibitory functions. However, the role of BASP1 in lung cancer is still unclear.

Brain metastases are common in NSCLC, and their biology is still poorly understood. EGFR mutation is significantly associated with lung cancer patients developing brain metastases 12, suggesting elevated EGFR signaling is important for brain metastasis. In this study, differ from the known function of BASP1 as a tumor suppressor, we identified that BASP1 was overexpressed in brain metastases and associated with poor outcomes. We investigated the function of BASP1 in metastatic lung cancer cells, focusing on the interaction between EGFR and BASP1, and searched for potential drugs to target the BASP1-EGFR axis to overcome TKI resistance in lung cancer.

## Materials and Methods

### Cell culture and *in vivo* selection of metastatic derivatives

Human lung adenocarcinoma cell lines CL1-0 (low invasiveness), F4 (high invasiveness), and Bm7 (high invasiveness) originate from the same lung cancer. All cell lines were tested and confirmed to be free of mycoplasma. Metastatic derivatives, including brain metastatic sublines, were obtained as previously described 13. PC9, A549, H1650, HCC827, and H1975 lung cancer cells were cultured in RPMI 1640 with 10% FBS, penicillin (P), and streptomycin (S). HEK293T and H2981 lung cancer cells were cultured in DMEM plus 10% FBS and 1% P/S. HCC827-GR8 is derived from HCC827 cells with long-term gefitinib treatment 14.

### Proteomics

Each membrane protein fraction isolated from the indicated lung cancer cell lines by the membrane protein enrichment kit was separated by SDS-PAGE and then subjected to in-gel enzymatic digestion. The tryptic peptides were identified by the linear ion trap-Fourier transform ion cyclotron resonance mass spectrometer (LTQ-FTICR MS, Thermo Electron) independently in duplicate 15. Identification of protein and label-free quantitative analysis were performed using MaxQuant 16 and MaxLFQ 17 software, respectively. A total of 233 proteins that exhibited at least a 2-fold increase in brain-metastatic cancer cells (Bm7 *vs*. F4) were identified.

### Immunohistochemistry staining

Two human lung cancer tissue arrays (LC10012 and LC10013) were purchased from US Biomax (62 adenocarcinoma samples and their corresponding adjacent normal tissues). BASP1 immunohistochemistry (IHC) staining was carried out as previously described 13 using rabbit human BASP1 antibody (ab103315; Abcam) and horseradish peroxidase-conjugated avidin-biotin complex (ABC) from the Vectastain Elite ABC Kit (Vector Laboratories, Burlingame, CA), and AEC chromogen (Vector Laboratories). The sections were counterstained with hematoxylin and mounted. IHC staining was scored by experienced histologists. The primary and metastatic specimens were obtained from the China Medical University Hospital (CMUH) in compliance with protocols approved by the CMUH IRB.

### Plasmids and shRNAs

The BASP1 plasmid RC201815 was purchased from OriGene (Rockville, MD) and used to construct the BASP1-GFP fusion expression plasmid. Lentiviral shRNAs targeting *BASP1* (clone E2: TRCN0000281253; clone H1: TRCN0000149347) and *C-CBL* (clone TRCN0000039727) were obtained from the National RNAi Core Facility (Institute of Molecular Biology, Genomic Research Center, Academia Sinica, Taiwan).

### Animal studies

Bm7 cells with stable luciferase expression (5 × 10^4^ cells) were injected intracardially into 6-8-week-old SCID mice (BioLASCO, Taiwan) and imaged by an IVIS Spectrum Imaging system (Xenogen, Hopkinton, MA, USA) under specific pathogen-free conditions as previously described 18. The incidence of tumor growth and the site of metastasis was quantified based on the luminescent signal at a given time point. For subcutaneous tumor models, 1 × 10^6^ cells in 150 μl PBS were subcutaneously injected into the right flank of six-week-old SCID mice. Tumor volume was calculated using the following equation:

tumor volume = length × width × width/2

SCID mice were subcutaneously implanted with H1975 lung cancer cells (1 × 10^6^). When H1975 tumors reached approximately 100 mm^3^, mice were randomized to receive vehicle (mock), afatinib, and a combination of afatinib (oral, 5 mg/kg daily for 5 days a week; AbMole BioScience) and arsenic trioxide (intraperitoneal injection (ip), 5 mg/kg three times a week; TTY Biopharm Company Limited) for 11 weeks. The dose of afatinib followed the previous report 19 and the dose of arsenic trioxide was adjusted for long term treatment from the previous study 20, 21. All animal experiments were carried out under protocols approved by the Institutional Animal Care and Use Committee of China Medical University and Hospital.

### Statistical analysis

Student's *t* test was applied for at least three independent biological replicates to calculate significance. The McNemar test and Fisher's exact test were applied for BASP1 IHC analysis, and the Wilcoxon test was applied to assess BASP1 expression in lung tumor and normal lung tissues in TCGA. Survival was assessed by the Kaplan-Meier method.

## Results

### BASP1 overexpression is associated with tumor progression and poor outcomes in lung adenocarcinoma

**Figure 1:**
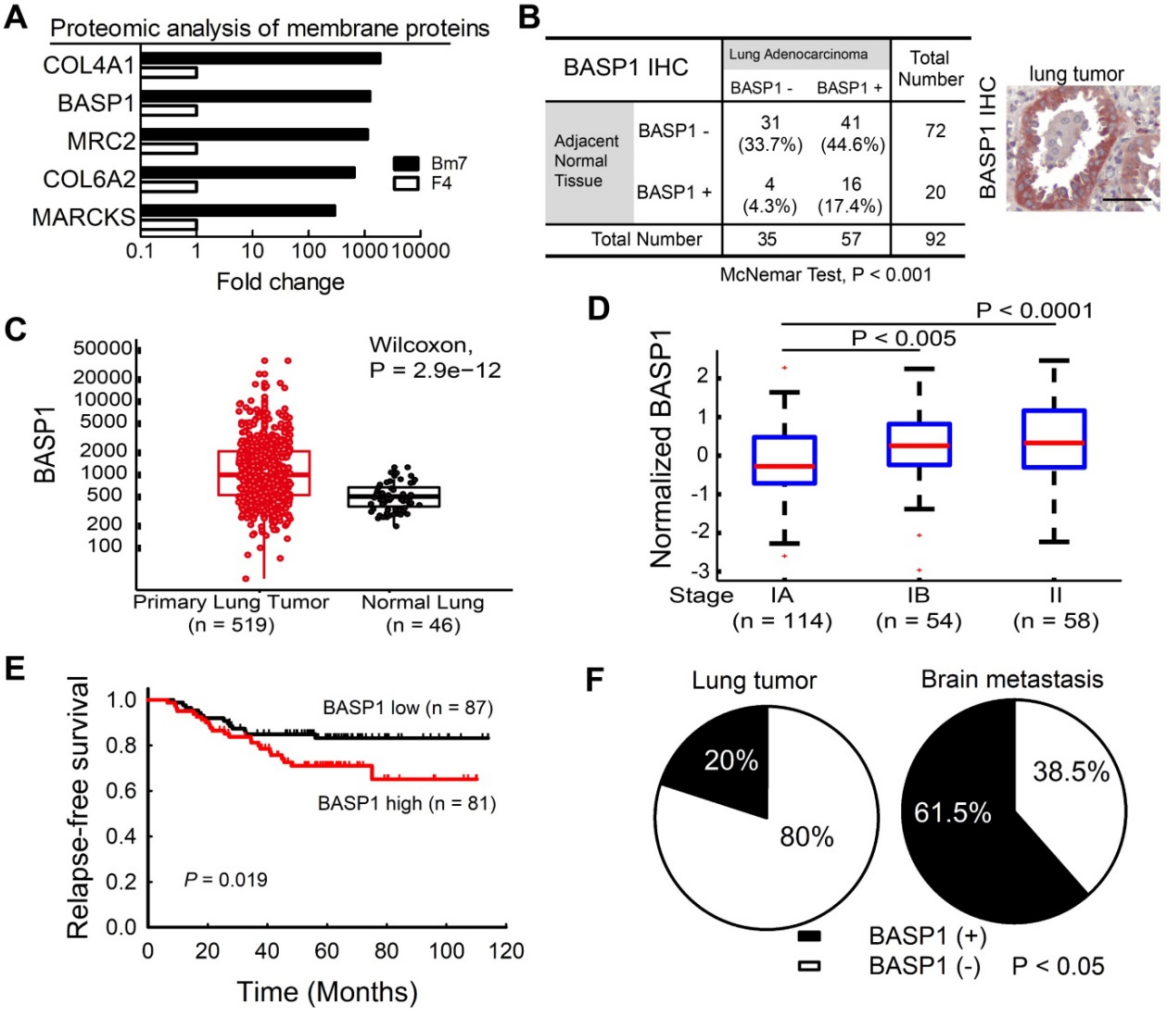
** Higher BASP1 expression correlates with poorer prognosis in lung adenocarcinoma patients. (A)** The levels of membrane proteins from established brain-metastatic subline Bm7 and parental F4 lung cancer cells were identified by liquid chromatography-mass spectrometry (LC/MS). COL4A1: collagen type IV alpha-1 chain; MRC2: C-type mannose receptor 2; COL6A2: collagen type VI alpha-2 chain. **(B)** IHC analysis of BASP1 in a human lung adenocarcinoma tissue array scored by staining intensity from 0 to 3+ by a histologist. A score of 0 to 1+, and 2 to 3+, indicate negative and positive staining of BASP1, respectively. Matched lung adenocarcinoma and adjacent normal tissues from the same patients were analyzed for the distribution of BASP1 staining by the McNemar method. Representative staining for BASP1 is shown (right). Scale bar, 50 μm. **(C)** Box plot of BASP1 expression in primary lung tumor samples (n = 519) and normal lung tissue samples (n = 46) from the TCGA LUAD dataset. Wilcoxon test. **(D)** Box plot of log_2_ (BASP1 expression) in stage IA (n = 114), stage IB (n = 54), and stage II (n = 58) primary lung tumor samples from the GSE31210 dataset. Student's *t*-test. **(E)** Kaplan-Meier survival analyses of lung adenocarcinoma patients with stage I and II disease from GSE31210; patients were divided into two groups (high or low gene expression) using the median level of BASP1 as the cutoff, and survival was analyzed with the log-rank test. **(F)** IHC analysis of BASP1 in clinical paraffin block specimens of primary lung tumors (n = 40) and brain metastasis tumor specimens (n = 13) of human lung adenocarcinoma from CMUH. The significant difference in IHC staining of BASP1 from the two groups was calculated by Fisher's exact test.

Membrane proteins are involved in signal transduction to coordinate intracellular pathways that promote tumor progression. To identify those that are involved in the aggressive phenotype of malignancy, we conducted comparative membrane proteomics between F4 parental lung cancer cells and their brain-metastatic counterparts (Bm7 cells) 13 by mass spectrometric analysis. The results from quantitative analysis identified the five proteins with the highest fold-change in expression in Bm7 cells relative to F4 cells (Figure 1A); these five proteins included two neuronal growth-associated proteins, BASP1 and MARCKS. The functions of MARCKS have been studied previously 22. Therefore, we focused on BASP1 for further investigation.

We further analyzed BASP1 expression in a human lung adenocarcinoma tissue microarray by IHC and showed that lung tumors exhibited higher BASP1 expression than adjacent normal lung tissues (*P <* 0.001; Figure 1B). Moreover, analysis of the lung adenocarcinoma dataset from The Cancer Genome Atlas (TCGA) also indicated significantly higher levels of BASP1 in lung cancers than in normal lung tissues (Figure 1C). Next, we investigated the gene expression profiles of lung adenocarcinoma from the GSE31210 dataset 23, 24. As shown in Figure 1D, patients in stages IB and II had significantly higher BASP1 levels than those in stage IA. Patients with high (above the median) expression of BASP1 also had decreased relapse-free survival compared to that in those with low BASP1 (Figure 1E). Similar negative effects of BASP1 on patient survival were confirmed in other public datasets, including the GSE11969 from Japan 25 and GSE30219 from France 26, and by Kaplan-Meier plotter 27 and PrognoScan, which is a database for meta-analysis of prognostic value of genes 28 (Figure S1A-D). Importantly, increased BASP1 expression was observed in 61.5% of brain metastases but only in 20% of primary lung tumors by IHC staining (Figure 1F). Taken together, these data indicate that BASP1 is associated with poor outcomes in patients with lung adenocarcinoma and that its expression is enriched after metastasis to the brain.

### BASP1 promotes lung cancer progression *in vitro* and *in vivo*

**Figure 2:**
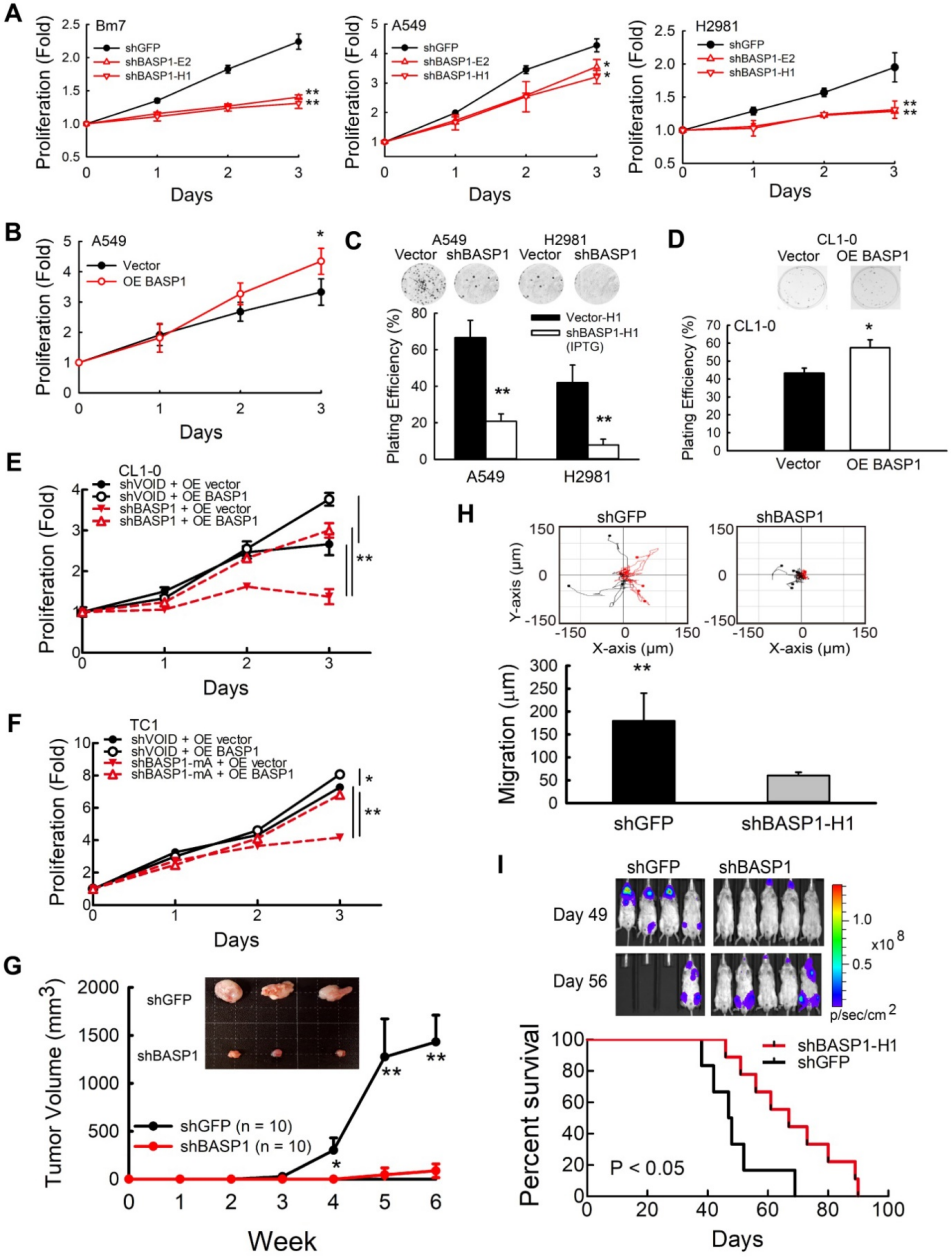
** BASP1 increases lung cancer cell growth and metastasis. (A)** The relative proliferation rate of control (shGFP) and *BASP1*-knockdown cells (shBASP1-E2 and shBASP1-H1) in Bm7 cells, A549 cells, and H2981 lung cancer cells was measured at the indicated time points by MTT assay. **(B)** Analysis of cell growth of A549 cells transfected with plasmids of BASP1 (OE BASP1) or vector alone. **(C)** The clonogenicity of BASP1-knockdown lung cancer cells (A549 and H2981) with IPTG-inducible shRNA was indicated by plating efficiency in a colony forming assay. Colonies were visualized by crystal violet staining of the cultures after 14 days. **(D)** The clonogenicity of CL1-0 lung cancer cells transfected with plasmids of BASP1 or vector alone. **(E)** Analysis of cell growth of control (shVOID) and *BASP1*-knockdown CL1-0 cancer cells overexpressing BASP1-GFP or control vector. **(F)** Analysis of cell growth of control and *BASP1*-knockdown (shBASP1-mA) TC1 mouse lung cancer cells overexpressing Basp1-GFP. **(G)** Control and *BASP1*-knockdown cells were injected subcutaneously into SCID mice (n = 10). Representative tumor images in the control and *BASP1*-knockdown groups are shown. **(H)** The migration rates of control and *BASP1*-knockdown cells were measured by time-lapse video microscopy in each group (top) and quantified (bottom). **(I)** Luciferase-expressing control or *BASP1*-knockdown cells were intracardially injected into SCID mice (n = 10 for each group). Representative images by IVIS from days 49 and 56 post injection are shown (left). The mouse survival time was monitored for 90 days. Survival was analyzed with the Kaplan-Meier method.

We first validated the specificity of the BASP1 antibody in *BASP1*-knockdown A549 and BASP1-overexpressing CL1-0 lung cancer cells with transient transfection of BASP1-GFP (Figure S2A). Western blotting showed multiple bands representing BASP1. The predicted molecular weight of BASP1 is 23 kDa, and oligomerization, protein modification, and unusual amino acid composition have been reported to contribute to its anomalous mobility in gel electrophoresis 29. Next, we assessed the role of BASP1 in tumorigenesis in *BASP1*-knockdown cells established by short-hairpin RNA (shRNA) targeting different regions of BASP1. The results indicated that depletion of BASP1 expression reduced proliferation in several lung cancer cell lines, including Bm7, A549, H2981, and PC9 cells (Figure 2A and Figure S2B). In contrast, overexpressing BASP1 (OE BASP1) increased the proliferative abilities of A549 cells (Figure 2B) and HCC827 lung cancer cells compared to those in control cells (dose-dependent increase; Figure S2C). Because BASP1 knockdown led to a significant reduction in cell numbers after several passages, we utilized an isopropyl thiogalactpyranoside (IPTG)-inducible shRNA knockdown system to better investigate the effects of BASP1 knockdown. In addition to lowering cell proliferation after IPTG induction (Figure S2D), BASP1 knockdown attenuated colony formation (Figure 2C). Compared to the control cells, BASP1-overexpressing CL1-0 cells, which contain low levels of BASP1, showed increased colony formation (Figure 2D). The rescue experiment showed that re-expression of BASP1 (GFP tagged; Figure S2E) increased the proliferation of *BASP1*-knockdown CL1-0 and control (shVOID) cells (Figure 2E). Additionally, transient transfection of mouse shRNA-resistant GFP-tagged BASP1 into mouse *BASP1*-knockdown TC1 lung cancer cells (Figure S2F) restored cell proliferation (Figure 2F). *In vivo*, SCID mice that received subcutaneous injection of *BASP1*-knockdown Bm7 (Bm7-shBASP1) cells had significantly smaller tumors than those injected with the Bm7-shGFP control cells (Figure 2G). Together, these results support the notion that BASP1 promotes the tumorigenesis of lung cancer cells *in vitro* and *in vivo*.

To determine whether BASP1 knockdown affects cell motility, we analyzed the migration ability of Bm7-shBASP1 cells by time-lapse microscopy. The migration distance of Bm7-shBASP1 cells was significantly decreased compared with that of the shGFP control (Figure 2H and Figure S2G). To further investigate the effects of BASP1 on promoting cancer metastasis, we injected luciferase-expressing Bm7-shGFP control or Bm7-shBASP1 cells intracardially into SCID mice to monitor the occurrence of metastasis by bioluminescence imaging (IVIS). Mice injected with Bm7-shBASP1 cells exhibited delayed metastasis to the brain, lungs, and bone and had longer survival times than those injected with Bm7-shGFP cells (Figure 2I). Notably, brain metastasis in Bm7-shBASP1 mice was delayed compared to that in the control mice (Figure 2I). Taken together, these results demonstrate that the expression of BASP1 can foster lung cancer metastasis.

### BASP1 promotes lung cancer progression by activating EGFR signaling

**Figure 3:**
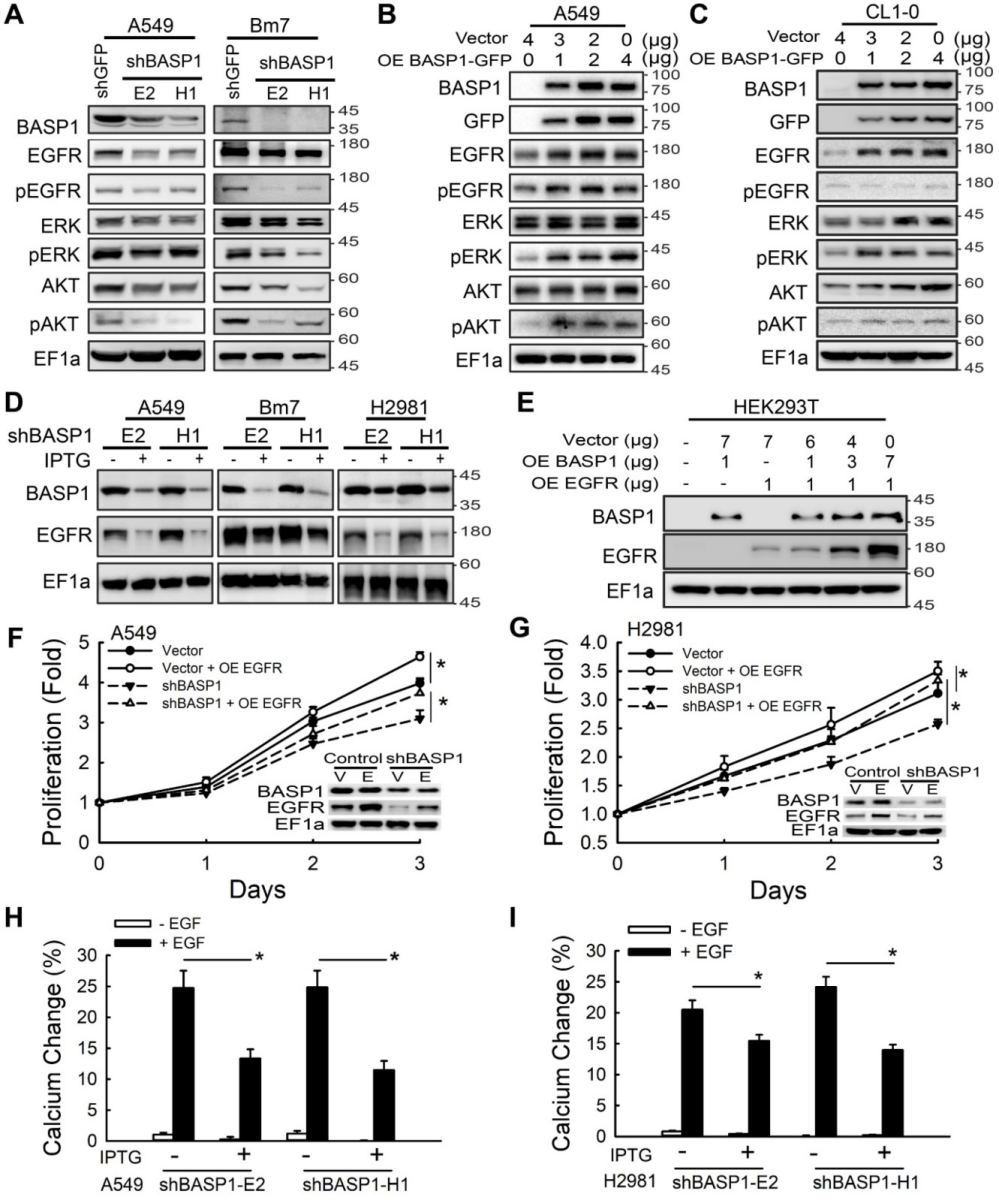
** BASP1 enhances EGFR signaling and EGFR protein expression to promote lung cancer progression. (A)** Lysates of BASP1-knockdown A549 and Bm7 lung cancer cells were subjected to immunoblotting with the indicated antibodies. **(B and C)** Western blot of EGFR signaling pathway proteins in A549 (B) and CL1-0 (C) lung cancer cells overexpressing BASP1-GFP. **(D)** Western blot of BASP1 and EGFR in lung cancer cell lines (A549, Bm7, and H2981) with IPTG-inducible shBASP1 expression. **(E)** Western blot of BASP1 and EGFR in HEK293T cells cotransfected with BASP1 and EGFR expression plasmids. The amount of transfected plasmids is shown above the blots. **(F and G) BASP1** knockdown-mediated inhibition of cell proliferation was rescued by EGFR overexpression. IPTG-induced BASP1 knockdown by shBASP1 in A549 (F) and H2981 (G) cells. Vector, control cells without IPTG induction. EGFR plasmids were transfected into lung cancer cells 2 days after IPTG induction and subjected to MTT assays to determine cell proliferation. **(H and I)** Intracellular calcium concentrations of control and IPTG-inducible BASP1 knockdown in A549 (H) and H2981 (I) lung cancer cells. Cells were serum starved for 4 hours and then treated with 50 ng/mL EGF. Data represent the mean ± SD. *P < 0.05, Student's *t*-test.

Aberrant activation of membrane RTKs is one of the critical regulatory nodes in the cancer signaling network 30. To investigate whether BASP1 activates membrane RTK signaling, we used a human phospho-RTK array to compare the relative signal intensities of phospho-RTKs between control and IPTG-induced *BASP1*-knockdown cells in two lung cancer cell lines. BASP1 knockdown inactivated the phosphorylation of several RTKs, but EGFR was the most inhibited RTK (Figure S3A-C). Given that aberrant EGFR signaling is a significant driver of lung cancer and that approximately 50% of Asian patients with NSCLC harbor EGFR mutations, we further investigated whether BASP1 regulates EGFR signaling. BASP1 knockdown in A549, Bm7, and PC9 cells inhibited the phosphorylation of not only EGFR but also ERK and AKT, both of which are downstream of EGFR signaling (Figure 3A and Figure S3D). The total EGFR protein levels in both EGFR wild-type (A549 and Bm7) and EGFR mutant (PC9) cells were also reduced. In contrast, ectopic expression of BASP1 in lung cancer cells increased the levels of endogenous EGFR, phospho-EGFR, phospho-ERK, and phospho-AKT (Figure 3B-C). Similar results were observed in the IPTG-inducible *BASP1*-knockdown system, in which EGFR expression was substantially reduced upon IPTG induction (Figure 3D and Figure S3E). In HEK293T cells with low endogenous BASP1 and EGFR, EGFR protein expression was increased in a dose-dependent manner with the transfection of fixed amounts of EGFR-expressing plasmids and increasing amounts of BASP1-expressing plasmids (Figure 3E). These results indicate that BASP1 facilitates EGFR protein expression. Moreover, cell growth was restored in EGFR-overexpressing *BASP1*-knockdown cancer cells (Figure 3F-G). These findings suggest that BASP1 promotes cell proliferation by increasing EGFR expression.

In addition to controlling cell proliferation, activation of the EGFR axis can activate intracellular calcium signaling to induce tumor cell migration 31. Thus, we asked whether BASP1 knockdown blocks intracellular calcium flux by attenuating EGFR signaling. Bm7 and H2981 lung cancer cells treated with EGF exhibited significantly elevated intracellular calcium concentrations compared with those without EGF treatment, whereas BASP1 knockdown significantly attenuated the intracellular calcium response to EGF stimulation, likely due to the downregulation of EGFR (Figure 3H-I and Figure S3F-H). Collectively, these findings suggest that BASP1 activates EGFR signaling to enhance cell proliferation and intracellular calcium signaling, which is essential for cell migration.

### BASP1 attenuates EGFR degradation

**Figure 4:**
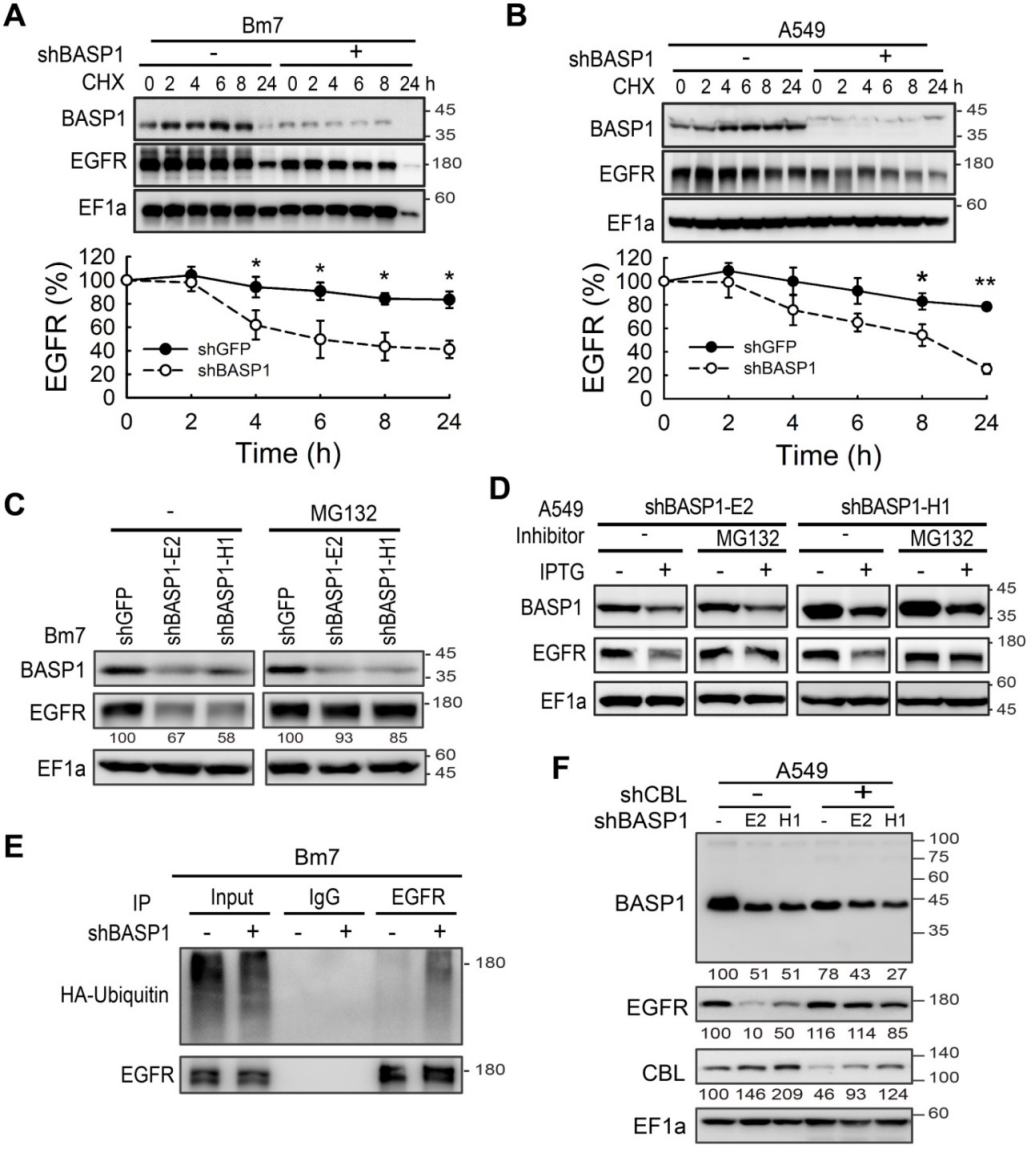
** BASP1 reduces ubiquitin-mediated EGFR degradation. (A and B)** *BASP1* knockdown enhanced EGFR protein degradation. Control and *BASP1*-knockdown Bm7 (A) and A549 lung cancer cells (B) were treated with 100 µM cycloheximide (CHX) for the indicated time periods. Western blot of BASP1 and EGFR. Relative EGFR expression was determined by measuring the EGFR band density from three independent experiments. Data represent the mean ± SD. *P < 0.05, student's *t*-test. **(C)** Control and *BASP1*-knockdown Bm7 lung cancer cells were cultured under starvation for 16 hours and then treated with EGF (50 ng/mL) for 2 hours before collecting cell lysates. Cells were treated with the proteasome inhibitor MG132 (5 µM) for 3 hours before EGF stimulation. **(D)** Control and IPTG-induced *BASP1* knockdown A549 lung cancer cells were cultured under starvation for 16 hours and then treated with EGF (50 ng/mL) for 2 hours before collecting cell lysates. Cells were treated with the proteasome inhibitor MG132 (5 µM) for 3 hours before EGF stimulation. **(E)** IPTG-induced shBASP1 of Bm7 cells with HA-ubiquitin overexpression followed by MG132 and EGF treatment for 2 hours. EGFR was immunoprecipitated from cell extracts using an EGFR antibody. **(F)** Western blot of BASP1, EGFR, and CBL in control and *BASP1*-knockdown cells transiently transfected with shRNA against *CBL*.

To understand the mechanism underlying BASP1-mediated expression of EGFR, we found that BASP1 knockdown did not affect the levels of endogenous EGFR mRNA (Figure S4A). Next, we examined EGFR protein degradation by cycloheximide (CHX) pulse-chase analysis. Knocking down BASP1 accelerated the degradation rate of EGFR (Figure 4A, 4B, Figure S4B), whereas treatment with proteasome inhibitor MG132 restored EGFR levels (stable knockdown in Figure 4C and Figure S4C; IPTG-induced knockdown in Figure 4D and Figure S4D). These results suggest that BASP1 protects EGFR proteins from undergoing proteasome-mediated degradation.

Because ubiquitin conjugation is essential for proteasomal protein degradation 32, we also investigated the effects of BASP1 on EGFR ubiquitination. Knocking down BASP1 increased the ubiquitination of immunoprecipitated EGFR (Figure 4E and Figure S4E). Next, we asked whether BASP1 reduced ubiquitination by the well-known EGFR E3 ubiquitin ligase CBL to affect EGFR degradation 33. As shown in Figure 4F, EGFR expression was rescued in A549 cells with both CBL and BASP1 knockdown (shCBL/shBASP1). These data suggest that BASP1 antagonizes CBL-mediated ubiquitination to increase EGFR stability.

### BASP1 coexists and interacts with EGFR in lipid rafts

**Figure 5:**
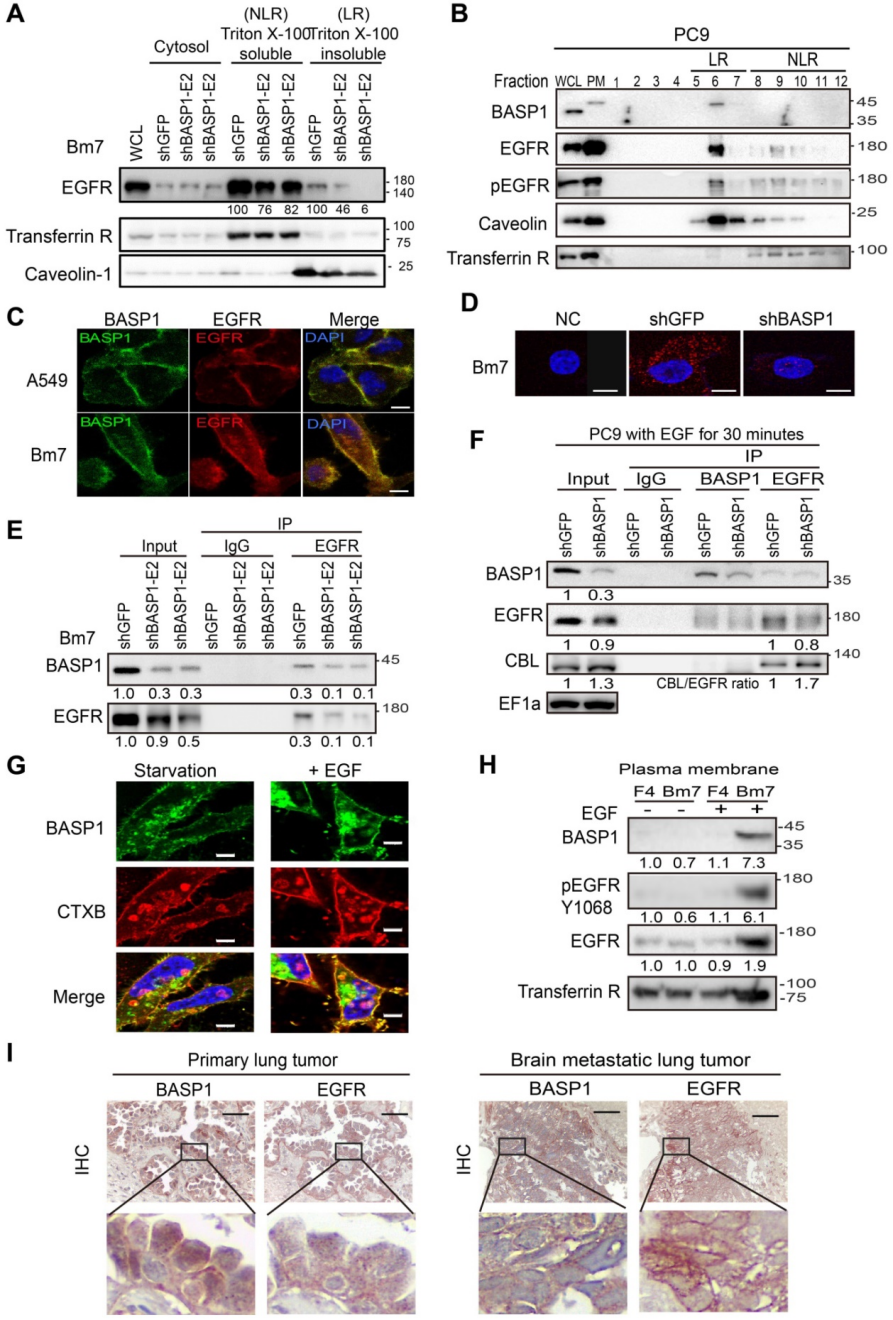
A positive feedback of BASP1 and EGFR. **(A)** Western blot analysis of membrane proteins from Triton X-100 soluble (nonlipid raft, NLR) and Triton X-100 insoluble (lipid raft, LR) fractions. Transferrin receptor, control of nonlipid raft; caveolin-1, control of lipid raft. **(B)** Western blot analysis of plasma membranes of PC9 cells with density gradient ultracentrifugation fractionation. PM, plasma membrane. Fraction is indicated by the collecting tube number from top to bottom after centrifugation. **(C)** Confocal microscopy of endogenous BASP1 and EGFR in lung cancer cells. Scale bar, 5 µm. **(D)** Proximity ligation assay for BASP1 and EGFR in Bm7 lung cancer cells (red fluorescence dots). Scale bar, 5 µm. **(E)** Coimmunoprecipitation of BASP1 and EGFR in Bm7 cells. **(F)** Co-IP analysis in PC9 cells treated with EGF for 30 min. **(G)** Confocal microscopy of BASP1-GFP expression (green) and CTXB staining in lipid rafts (red) in CL1-0 cells transiently transfected with BASP1-GFP plasmids and treated with EGF for 10 minutes. Scale bar, 7 µm. **(H)** Western blot analysis of the plasma membrane fraction of F4 and Bm7 cells treated with EGF (50 ng/mL) for 15 min. **(I)** Representative images of BASP1 and EGFR IHC staining in serial sections of clinical paraffin block specimens of primary lung tumor and brain metastasis tumor specimens from different lung cancer patients. Scale bar, 50 µm.

EGFR endocytic trafficking plays an important role in modulating the degradation and termination of EGFR signaling 34. High-dose EGF is known to induce EGFR internalization via lipid raft-associated or clathrin-independent mechanisms, whereas low-dose EGF is reported to stimulate EGFR endocytosis through a clathrin-dependent mechanism 35. To determine which of these mechanisms facilitates EGFR degradation in *BASP1*-knockdown cells, we examined the levels of EGFR at high and low doses of EGF in these cells. EGFR degradation was observed at high (50 ng/mL) but not low (5 ng/mL) doses of EGF after 90 min (Figure S4F), suggesting that BASP1-mediated EGFR stabilization occurs via the lipid raft-associated pathway. To validate this, we separated plasma membranes into soluble (nonlipid rafts, NLR) and insoluble (lipid rafts, LR) fractions using transferrin receptor and caveolin-1 as indicators for the nonlipid raft and lipid raft fractions, respectively 36. Compared with the control cells, BASP1 knockdown markedly decreased EGFR expression in the lipid raft but only moderately decreased EGFR expression in the nonlipid raft fraction (Figure 5A). Density gradient ultracentrifugation revealed that BASP1 coexisted with EGFR/phospho-EGFR proteins in the same lipid raft fractions (fractions 5-7; Figure 5B). These findings suggest that BASP1 and EGFR are colocalized in lipid rafts.

Next, we asked whether BASP1 directly interacts with EGFR. Using confocal microscopy, we showed that BASP1 and EGFR colocalized on the cell membrane in A549 and Bm7 cells (Figure 5C), and this was validated by an *in situ* proximity ligation assay 37 (Figure 5D). A coimmunoprecipitation (co-IP) assay also demonstrated BASP1-EGFR interaction in Bm7 and H2981 cells (Figure 5E and Figure S4G) as well as on the plasma membrane of CL1-0 cells that were transfected with BASP1-GFP plasmids (Figure S4H).

Because our results above (Figure 4F) indicated that BASP1 prevents EGFR from CBL-mediated ubiquitination, we examined CBL expression in EGFR immunoprecipitates. The levels of CBL pulldown were higher in EGFR immunoprecipitates from *BASP1*-knockdown cells than in those from control cells (Figure 5F). These results suggest that BASP1 may influence CBL binding to EGFR to prevent its ubiquitination.

### Reciprocal regulation of BASP1 and EGFR signaling in lung cancer cells

Since BASP1 colocalized with EGFR in the plasma membrane, we asked whether EGFR signaling regulates BASP1 localized in the plasma membrane. By using cholera toxin B-subunit (CTXB) as a marker of lipid rafts, we found that EGF (50 ng/ml) stimulation increased the amount of BASP1 to localize to the lipid rafts (Figure 5G). The increase in BASP1 occurred within 5 min after EGF stimulation (Figure S4I-J). In contrast, EGFR TKI erlotinib abrogated the increase in membrane-associated BASP1 after EGF stimulation (Figure S4K). EGF stimulation increased the levels of membrane-associated BASP1, EGFR, and phosphorylated EGFR in brain-metastasis sublines and Bm7 cells but not in the F4 parental cells, suggesting that dual-high levels of BASP1 and EGFR occurred in brain-metastatic cells under EGF stimulation (Figure 5H). Analysis of primary and brain-metastatic tumor specimens from patients with NSCLC indicated that BASP1-positive samples also had strong EGFR staining. Notably, the intensity of membrane EGFR staining was stronger in metastatic brain tumors than in primary lung tumors (representative images shown; Figure 5I). All these data suggest that BASP1-abundant lung cancer cells facilitate EGFR signaling amplification than BASP1-deficient cells.

### Inhibition of BASP1 sensitizes lung adenocarcinoma cells to EGFR inhibitors

**Figure 6:**
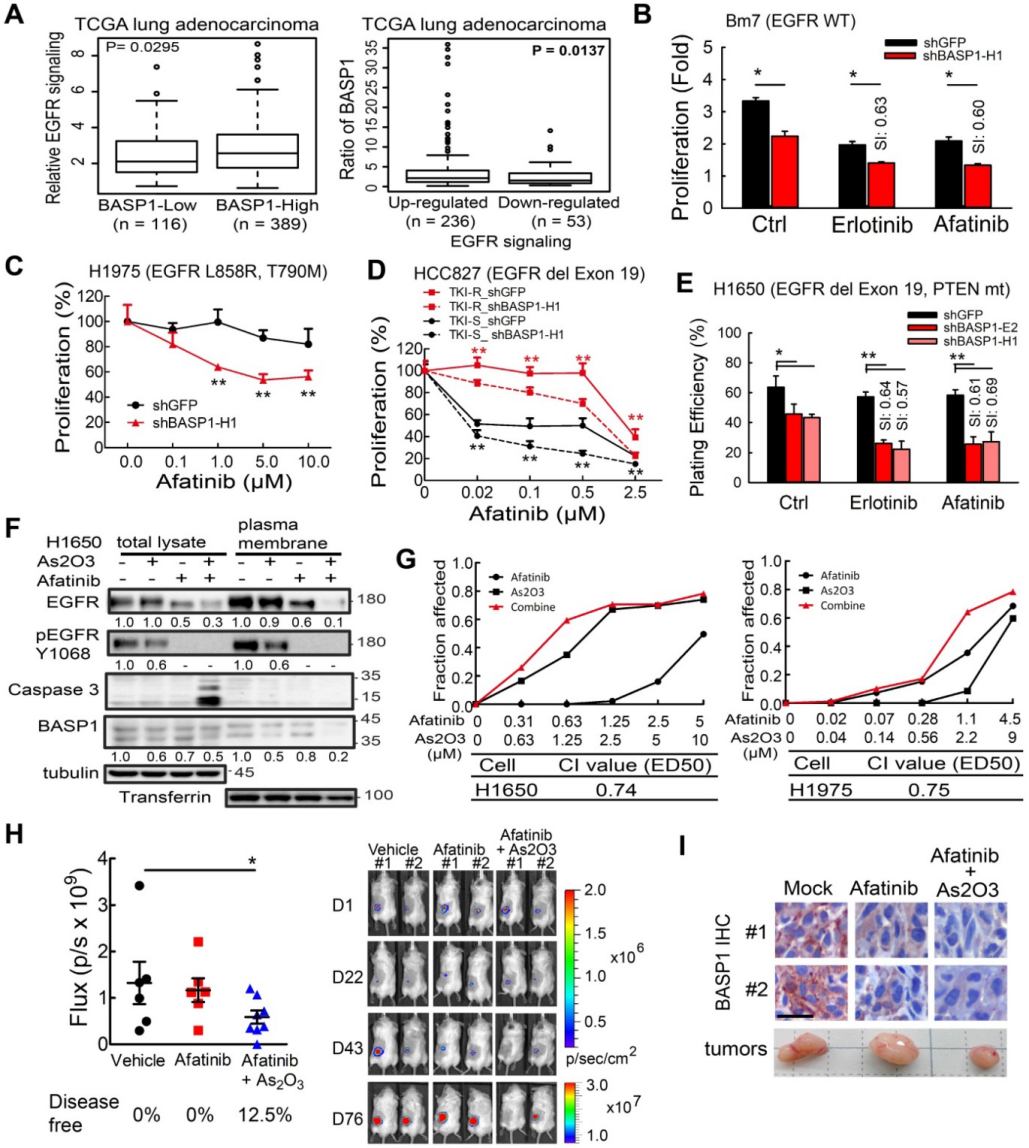
** The effects of BASP1 reduction in lung cancer cells treated with EGFR inhibitors. (A)** Relative EGFR signaling in the TCGA lung adenocarcinoma dataset classified by *BASP1* expression. BASP1-low and BASP1-high, *BASP1* expression in tumors was lower or higher than that in normal tissues, respectively (left). The relative expression of BASP1 in the TCGA lung adenocarcinoma dataset with upregulated or downregulated EGFR signaling gene signatures (right). *P* values, Welch's t-test. **(B)** Cell proliferation of Bm7 cells treated with TKI by MTT assay. SI, sensitization index. Erlotinib, 20 µM. Afatinib, 10 µM. **(C)** Cell proliferation of H1975 cells treated with afatinib for 3 days by MTT assay.** (D)** Cell proliferation of TKI-sensitive (TKI-S) HCC827 and TKI-resistant (TKI-R) HCC827-GR8 cells treated with afatinib. Cells were transiently transfected with shRNA against BASP1 and then treated with different concentrations of EGFR TKIs for 3 days. IC50 of shGFP and shBASP1 in TKI-R cells: 2.12 and 0.82 μM; IC50 of shGFP and shBASP1 in TKI-S cells: 0.063 and 0.005 µM. **(E)** Plating efficiency of H1650 lung cancer cells treated with EGFR TKIs by colony formation assay. Erlotinib, 5 µM. Afatinib, 1 µM. **(F)** Western blot analysis of indicated proteins from H1650 lung cancer cells treated with As_2_O_3_ and afatinib for 24 hours. **(G)** Synergistic therapeutic effects of As_2_O_3_ and afatinib in H1650 and H1975 cells by MTT assay. Combination index (CI) analysis (bottom). **(H)** Comparison of bioluminescence signals of tumors from SCID mice bearing subcutaneous H1975 lung cancer cells treated with afatinib and As_2_O_3_ on day 76. Vehicle (N=6); afatinib (N=6); afatinib + As_2_O_3_ (N=8). Representative images of tumor signals from the IVIS system (right). **(I)** IHC analysis of BASP1 in H1975 lung tumors from mice models. Two individual tumors. Scale bar, 25 µm.

Analysis of a dataset of lung adenocarcinoma specimens from the TCGA database indicated that samples with high BASP1 expression exhibited significantly elevated EGFR signaling (Figure 6A, left). Alternatively, when the samples were separated into upregulated and downregulated EGFR signaling, elevated BASP1 expression was observed in the upregulated EGFR signaling group (Figure 6A, right). These findings suggest that BASP1 and EGFR may form a positive feedback loop to enhance EGFR signaling.

Next, we evaluated the effects of BASP1 knockdown on EGFR inhibitors in different lung cancer cell lines that included EGFR-mutant and EGFR-WT cells. In EGFR wild-type cells, erlotinib or gefitinib treatment showed similar results to reduce the cell survival of BASP1 knockdown A549 cells in decreased IC_50_ values compared to control cells (Figure S5A-B). Moreover, BASP1 knockdown rendered those cells more sensitive to erlotinib, and afatinib compared with control cells (Figure 6B and Figure S5C) with sensitization index (SI) values of ~0.6, indicating synergistic cell killing effects 38. Cell proliferation assays indicated that BASP1 knockdown significantly improved the response of H1975 TKI-resistant cells, which harbor the EGFR T790M mutation, to afatinib (Figure 6C). Transiently transfection of shRNA against BASP1 induced higher cytotoxicity than transfection of shRNA control when combined with afatinib in both TKI-sensitive HCC827 and TKI-resistant HCC827-GR8 cells, as indicated by the significantly decreased IC_50_ values (Figure 6D). We examined the long-term effects of drug treatment in *BASP1*-knockdown and control cells by clonogenic assays. H1650 cells harbor the exon 19 deletion but develop resistance to TKIs due to the loss of the *PTEN* tumor suppressor 39. Erlotinib or afatinib alone had little or no effect on H1650 cells compared with the DMSO control (Figure 6E). In contrast, BASP1 knockdown markedly increased the cell killing effects of erlotinib and afatinib in H1650 cells, with SI values between 0.57 and 0.69 (Figure 6E and Figure S5E). Similar results were observed for the H1975 TKI-resistant and HCC827 TKI-sensitive (Figure S5D, S5F, and S5G) cells. These results indicate that targeting BASP1 may be a promising strategy to overcome lung cancers with different resistance mechanisms to EGFR TKIs.

To identify agents that can suppress BASP1 expression, we screened a library of traditional Chinese medicines for small compounds that target BASP1 and identified arsenic trioxide (As_2_O_3_), which has been used in the treatment of acute promyelocytic leukemia 40, as it decreased BASP1 proteins in CL1-0 and H1975 cells harboring wild-type and mutant EGFR, respectively (Figure S6A). We then evaluated the combination of As_2_O_3_ and TKI and showed that it substantially reduced the protein levels of BASP1, EGFR, and phospho-EGFR (Y1068) in the plasma membrane (Figure 6F). As_2_O_3_ plus afatinib also induced more apoptosis than either agent alone, as indicated by caspase 3 cleavage (Figure 6F), and synergistically reduced the cell proliferation of TKI-resistant H1650 and H1975 cells (CI value < 1; Figure 6G). Similar results were found for the EGFR wild type CL1-0 cells treated with As_2_O_3_ plus afatinib (Figure S6B). A synergistic effect in the cells treated with As_2_O_3_ plus erlotinib or osimertinib (AZD9291) was detected in EGFR mutant lung cancer cells (Figure S6C-D).

To evaluate the therapeutic effect of the combination of As_2_O_3_ and afatinib *in vivo*, we performed preclinical tumor models for lung cancer with EGFR mutant in the context of evaluating the add-on effect of As_2_O_3_ in afatinib treatment in SCID mice because treatment with As_2_O_3_ alone in SCID mice bearing H1975 tumors showed similar results in tumor volume and body weight as control group (Figure S6E-F). As shown in Figure 6H, mice treated with the combination of As_2_O_3_ and afatinib had significantly lower IVIS signals compared with the untreated control group, but afatinib alone had no inhibitory effect. A similar trend was observed in measured tumor volume that combo drugs group had smaller tumor volume compared to the control group (Figure S6G). Moreover, mice treated with the combo drugs for long-term (11 weeks) were still in healthy condition without symptoms of weight loss (Figure S6H). Notably, after treatment, 12.5% of mice in the combination group had tumors that disappeared and were disease-free (Figure 6H, right). Moreover, the staining intensity of BASP1 in tumors from mice treated with combination therapy was lower than that from the untreated control or treatment with afatinib alone (Figure 6I). Altogether, these findings demonstrate the synergistic therapeutic effects of combining As_2_O_3_ and TKIs, such as afatinib and erlotinib, in lung cancer cells and suggest a potentially effective approach to overcoming the acquired resistance of EGFR-mutant and EGFR wild-type lung cancer cells to EGFR TKIs.

## Discussion

**Figure 7:**
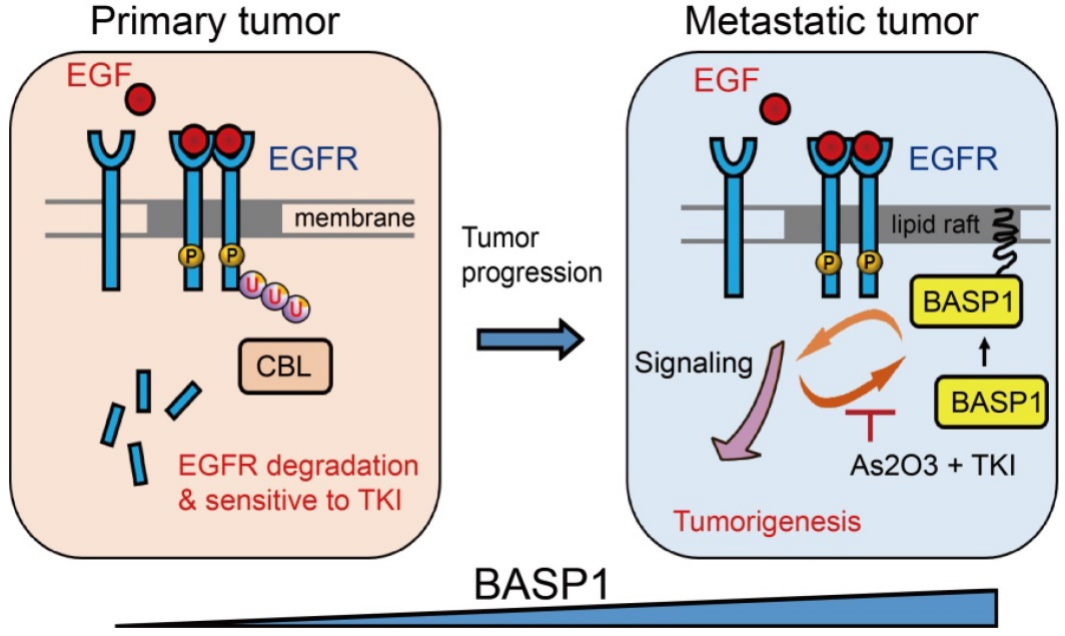
Proposed mechanism by which BASP1 promotes tumorigenesis through a positive regulatory loop of EGFR signal activation on the cell membrane.

In this study, we revealed a novel mechanism of BASP1 in promoting lung cancer malignancy (Figure 7): BASP1 enriches EGFR in lipid rafts and enhances EGFR signaling by reducing CBL-dependent EGFR ubiquitination. In turn, activation of EGFR signaling forms a positive feedback loop by recruiting more BASP1 to lipid rafts. Furthermore, BASP1 decreases the drug sensitivity of lung cancer cells treated with EGFR tyrosine kinase inhibitors (TKIs) (erlotinib and afatinib). Lastly, arsenic trioxide and EGFR TKIs reduce BASP1 expression and induce a synergistic inhibitory effect in lung cancer cells with acquired resistance to EGFR inhibitors.

BASP1 is known to localize in nucleus and harbor regulatory roles of gene transcription in cancer cells 41. In acute and chronic lymphocytic leukemia, BASP1 expression is downregulated 42, 43, suggesting a tumor suppressive role of BASP1. However, our findings demonstrated that most BASP1 exists in the cytosol and lipid rafts to promote cell survival of malignant lung cancer cells. We showed that activation of EGFR signaling can stimulate BASP1 to quickly locate in lipid raft; furthermore, the interplay between BASP1 and EGFR can strengthen the oncogenic signaling and overcome the resistance to EGFR inhibitors. It would be of interest to further investigate whether the functions of BASP1 dependent on its cellular distribution can be applied in other cancer types and which factors affect BASP1 location to have such different cell responses.

RTKs can promote tumor progression by cooperating multiple receptors to drive extensive oncogenic signaling, known as RTK co-activation network, and are highly dynamic regulation upon quickly adapting to RTK blockade 33. Currently, the basic strategy to disrupt this RTK co-activation network is through the combinations of selective RTK inhibitors or broadly TKIs to block several RTKs simultaneously 44, 45. We found that BASP1 knockdown can influence the signaling of several membrane RTKs, including EGFR, HER2, AXL, and hepatocyte growth factor receptor (HGFR)/c-MET by phospho-RTK array screening (Figure S3B). By focusing on the critical RTK in lung cancer, EGFR, we demonstrated that positive feedback between BASP1 and EGFR amplified the signaling in the lipid raft. Lipid rafts have been implicated in a variety of cellular processes, including signaling transduction of RTK 46, AXL 33 and c-MET 47 RTKs, by contributing to the pathophysiology of lung cancer and the acquired resistance to EGFR TKI. Given that aggregation of lipid rafts activated c-MET and its downstream signaling in NSCLC cells in radiation resistance 48, lipid rafts were a critical place to conduct the c-MET signaling. Indeed, our study revealed that targeting BASP1 interrupted the RTK co-activation and further lead to the lethal effects of lung cancer cells treated with RTK inhibitors. Further studies are still required to explore whether BASP1 can regulate other RTKs through similar positive feedback, like EGFR, in the lipid raft for cancer progression and acquired TKI resistance.

Our studies showed that *BASP1* knockdown decreased calcium influx in lung cancer cells treated with EGF, suggesting that BASP1 enhanced EGFR signaling and calcium influx to increase cell migration. Therefore, simultaneously blocking *BASP1* and EGFR signaling with EGFR tyrosine kinase inhibitors were expected to suppress the metastasis of lung cancer cells by suppressing calcium influx. Several studies have similar results showing that activation of EGFR enhanced calcium flux and cell migration 49, 50. In contrast, it has been reported that EGFR inhibitor gefitinib activated calcium release from the endoplasmic reticulum to suppress the growth of colorectal cancer cells 51. Although it seems to be opposite to the mechanism of our findings, that study was through an EGFR-independent manner. Thus, EGFR inhibitors may have different roles in regulating the intracellular influx by either on-targeting or off-targeting effects. Moreover, the different effects of calcium release might depend on the cancer cell types. The overall impact of EGFR inhibitors on intracellular calcium regulation in lung cancer cells is required to be investigated further.

EGFR TKIs have shown remarkable effects in the treatment of NSCLC with activating mutation of EGFR; however, acquired resistance eventually develops via the generation of new resistant mutations, even with the application of new-generation TKI inhibitors. Strategies other than inhibitors, including degradation of EGFR, may help to resolve this critical issue. As_2_O_3_, an ancient Chinese medicine, is a clinical drug for the treatment of acute promyelocytic leukemia by inducing degradation of oncogenic PML-RARα fusion proteins that result from t(15;17) translocation 52. Several studies have shown that As_2_O_3_ induces cell cycle arrest, cell apoptosis, DNA damage, and reactive oxygen species production in several types of cancers, including lung cancer 53. As_2_O_3_ has been used in the treatment of patients with acute promyelocytic leukemia with central nervous system relapses 54. Clinical studies showed that median arsenic levels in cerebrospinal fluid (CSF) were at 17.7% of the plasma levels, which was at therapeutically meaningful levels 55. Notably, it showed that a combination of arsenic trioxide and mannitol could increase the CSF arsenic concentration to ∼99.7% of those in the paired blood samples 56. Osimertinib is a third-generation, irreversible EGFR-TKI that potently and selectively inhibits both EGFR-TKI-sensitizing mutations (EGFR exon 19 and 21 mutations) and the T790M resistance mutation 57. It has recently been approved in the USA and Europe as a first-line treatment for advanced NSCLC patients with EGFR mutant and T790M mutation. Importantly, osimertinib is active to penetrate the blood-brain barrier to treat brain-metastasis lung cancer patients 58. Drugs with a suitable delivery system, such as using blood-brain barrier-penetrating codelivery liposomes could enhance the efficiency of therapy in lung cancer patients with brain metastases 59. Thus, combined therapy of arsenic trioxide and osimertinib may have potential benefits to treat lung cancer patients with brain metastases.

A recent study reported that the combination of As_2_O_3_ and EGFR inhibitors showed a synergistic anticancer activity through inhibition of DNA double-strand break repair mediated by EGFR 60. Similarly, our findings showed that As_2_O_3_ reduced BASP1 expression and induced synergistic effects to kill lung cancer cells when combined with EGFR TKIs. These results suggested that blockage of the positive feedback loop between BASP1 and EGFR is a potential treatment strategy for lung cancer acquired resistance to EGFR TKI inhibitors. Our findings may provide a molecular rationale for clinical trials. Also, our results revealed that coadministration of As_2_O_3_ and EGFR TKIs sensitized drug effects of lung cancer cells regardless of EGFR mutation status, which may expand the beneficial application of EGFR TKIs in lung cancer patients with amplified wild-type EGFR.

## Supplementary Material

Supplementary figures and tables.

## Abbreviations

As_2_O_3_: arsenic trioxide
BASP1: brain acid soluble protein 1
CHX: cycloheximide
co-IP: coimmunoprecipitation
CTXB: cholera toxin B-subunit
EGFR: epidermal growth factor receptor
GAP43: growth-associated protein 43
IPTG: isopropyl thiogalactpyranoside
LR: lipid rafts
MARCKS: myristoylated alanine rich protein kinase C substrate
NLR: nonlipid rafts
NSCLC: non-small-cell lung cancer
shRNA: short-hairpin RNA
TKIs: tyrosine kinase inhibitors
TCGA: The Cancer Genome Atlas
