# Supplementary material for: Targeting positive feedback between BASP1 and EGFR as a therapeutic strategy for lung cancer progression
Source: Theranostics. 2020 Aug 29;10(24):10925–39. doi: 10.7150/thno.49425 (PMC7532684; doi:10.7150/thno.49425)
Supplement: Supplementary file 1 — Supplementary figures and tables. [file thnov10p10925s1.pdf]

Supplemental Information

Targeting positive feedback between BASP1 and EGFR as a therapeutic strategy for lung cancer progression

Ching-Chan Lin, Yu-Kai Huang, Chia-Fong Cho, Yu-Sen Lin, Chia-Chien Lo, Ting-Ting Kuo, Guan-Chin Tseng, Wei-Chung Cheng, Wei-Chao Chang, Tzu-Hung Hsiao, Liang-Chuan Lai, Jin-Yuan Shih, Yu-Huei Liu, K.S. Clifford Chao, Jennifer L. Hsu, Pei-Chih Lee, Xian Sun, Mien-Chie Hung, Yuh-Pyng Sher

Includes:

1. Supplementary Methods
2. Supplementary References
3. Supplementary Figures

Supplementary Methods

Time-lapse Migration and Colony Formation Assay

Time-lapse migration assay was conducted as previously described [1]. Briefly, the migration distance of cells on collagen-coated dishes in serum-free media was traced using CCD video cameras (AxioCam MRm, Zeiss, Jena, Germany) and Track Point function of Image J software (NIH, Bethesda, MD, USA). Colony formation assay was performed as previously described [2]. The percentage of plating efficiency was calculated by dividing the number of colonies formed by the number of cells seeded and multiplied by 100.

IPTG-inducible shRNA System

To generate IPTG-inducible shBASP1 expression cell lines, the region of BASP1 targeted by shRNA was inserted into the pLAS1w.3xLacO vector. IPTG (500 μM) was used to induce the expression of shBASP1. BASP1 levels were detected by immunoblotting with BASP1 antibody.

Real-time PCR

Total RNA was extracted from tissues or cells with TRIzol (Invitrogen) and reverse

transcription performed with oligo dT primers according to the manufacturer's instructions. Real-time PCR was carried out in the LightCycler® 96 Real-Time PCR System with Universal Probe Library (Roche Diagnostics). Primers (5´ to 3´):

Human BASP1 forward: TGGATCTCAATGCCAATCCT

Human BASP1 reverse: GAGAATGTTTGTCACTCCCAAAA;

EGFR forward: GCCTTGACTGAGGACAGCA

EGFR reverse: TTTGGGAACGGACTGGTTTA;

Relative levels of human BASP1 and EGFR gene expression were determined using human GAPDH as the endogenous control. All reactions were carried out in triplicate.

Immunoprecipitation

Immunoprecipitation was performed as previously described [3]. Briefly, 500 μg cell lysates were mixed with 1 μg antibody for each reaction. The mixture was rotated at 4 °C overnight followed by the addition of 50 μL of protein A/G sepharose slurry. After rotation for another 4 h, beads were collected and washed twice with PBS containing 1% NP-40. Immunoprecipitates were resolved by SDS-PAGE followed by Western blotting with the indicated antibodies.

3-(4,5-dimethylthiazol-2-yl)-2,5-diphenyltetrazolium bromide (MTT) assay

MTT assays were conducted as previously described to measure cell proliferation [1]. Briefly, cells were incubated with 100 μL of MTT reagent (0.5 mg/mL) in 96-well plates for 4 h. The solubilized formazan was quantified at 570 nm using a spectrophotometer.

Immunofluorescence and *in situ* proximity ligation assay (PLA)

Lung cancer cells were fixed with 4% paraformaldehyde for 30 min and stained with primary antibody against BASP1 (ab103315; Abcam) or EGFR (MA5-13070; Thermo Fisher Scientific) overnight followed by incubation with FITC-conjugated mouse IgG conjugated or rhodamine-conjugated rabbit IgG secondary antibody for 1 hour. The slides were covered with DAPI (4´,6-diamidino-2-phenylindole) mounting medium (DUO82040; Sigma-Aldrich) and visualized using a fluorescent microscope with DAPI/FITC/rhodamine triple-pass filters. PLA was performed using the Duolink ***in situ***-Fluorescence (Sigma-Aldrich) according to the manufacturer's instructions.

Lipid raft isolation

Lipid raft membrane fractions were isolated using Triton X-100 extraction method or OptiPrep density gradient centrifugation assay as previously reported with modifications [4, 5]. Briefly, for Triton X-100 extraction method, cells were collected with TNE buffer (20 mM Tris-HCl, 150 mM NaCl, 2 mM EDTA, pH 7.4, and 2 mM sodium orthovanadate with protease inhibitors) and subjected to sonication. After centrifugation at 12,000 rpm for 20 min at 4°C, supernatants were collected as cytosolic fractions. Pellets were solubilized in TNE buffer with 1% Triton X-100 and centrifugation. The soluble fractions in the supernatant were collected and pellets were lysed with TNE buffer with 1% SDS and collected as insoluble fractions. For OptiPrep density gradient centrifugation assay, cells were collected with homogenization buffer (150 mM NaCl, 20 mM Na_2HPO_4, 2 mM NaH_2PO_4, 20% v/v glycerol, and 2 mM sodium orthovanadate with protease inhibitors, pH 7.4) and subjected to sonication. After centrifugation at 12,000 rpm for 20 min at 4°C, the supernatants were subjected to centrifugation at 32,000 rpm (Beckman-Coulter Optima L-90K ultracentrifuge with a SW90Ti rotor) for 90 min to collect the total plasma membrane. The pellets were solubilized in TNE buffer (20 mM Tris-HCl, 150 mM NaCl, 2mM EDTA, pH 7.4, and 2 mM sodium orthovanadate with protease inhibitors) containing 1% Triton X-100 and adjusted to a 40% final density by using OptiPrep density gradient medium (AXS-

1114542; Alere Technologies AS, Oslo, Norway). Samples were overlaid with a discontinuous 30%/5% OptiPrep density gradient and centrifuged at 38,000 rpm for 16 h at 4°C (SW41Ti rotor). Fractions were gently removed from the top of the gradient in 1-mL fractions. All fractions were then separated by SDS-PAGE and analyzed by Western blotting.

Intracellular calcium signaling

Intracellular calcium concentration was measured by imaging the fluorescence of the Ca^{2+} indicator dye, fluo-4 (Fluo-4 NW Assay Kit, #F36205; Thermo Fisher Scientific). Briefly, cells were grown overnight in 96-well black clear flat bottom polystyrene plates (Costar) at 37 °C with 5% CO_2. The growth medium was removed, and the cells were incubated with 100 µl dye-loading solution (4 µM fluo-4/AM) at 37 °C with 5% CO_2 incubator for 30 min and then at room temperature for another 30 min. After loading, cells were washed with PBS and fluorescence detection performed at room temperature.

Bioinformatics analysis

For correlation analysis between BASP1 and activation of EGFR signaling in

clinical lung cancer specimens, the expression profiling of lung adenocarcinoma samples (n = 505) and normal samples (n = 19) was downloaded from the Cancer Genome Atlas (TCGA). EGFR-upregulated signaling gene set (n = 18) was compiled from Ma et al. [6] and Amit et al. [7]; EGFR-downregulated signaling gene set (n = 25) was compiled from Amit et al. [7]. The samples were divided into two groups: upregulated EGFR signaling (n = 236) and downregulated EGFR signaling (n = 53) based on the following criteria: 1) upregulated EGFR signaling (n = 412) included samples with an average expression value of EGFR-upregulated signaling gene set of 1.5 times greater than that of all normal samples; 2) downregulated EGFR signaling (n = 229) included samples with an average expression value of EGFR-downregulated signaling gene set of 1.5 times less than that of all normal samples; 3) Samples (n = 176) that met both criteria were excluded for further analysis. The ratio of cancerous to normal *BASP1* was calculated in EGFR signaling up-regulated and down-regulated groups.

Conversely, tumor tissues with *BASP1* expression lower than the mean of normal samples were belong to '*BASP1* low group' (n=116) and those with *BASP1* expression higher than the mean of normal samples were divided into '*BASP1* high group' (n=389). The EGFR signaling gene set (n=43) was composed of both EGFR upregulated

signaling gene set. The average ratio of EGFR signaling gene in cancerous to normal tissues was calculated in BASP1-low and -high groups.

Key resources table

Antibodies		
BASP1 (for WB)	Abcam	Cat# ab103315, RRID:AB_10710130
BASP1 (for IHC)	Sigma	Cat# HPA045218 RRID: AB_2679263
BASP1 (for Immunofluorescence)	Abcam	Cat#ab101855 RRID: AB_10711377
EGFR (for IHC)	Abcam	Cat# ab52894 RRID: AB_869579
EGFR (for Immunofluorescence)	Thermo Fisher Scientific	Cat# MA5-13319 RRID: AB_10985841
EGFR (for Immunofluorescence)	Santa Cruz	Cat# sc-03 RRID: AB_631420
EF1α	Millipore	Cat# 05-235, RRID:AB_309663
EGFR	Santa Cruz Biotechnology	Cat# sc-03, RRID:AB_631420
phospho-EGFR (Tyr1068)	Cell Signaling Technology	Cat# 3777, RRID:AB_2096270
ERK	Cell Signaling Technology	Cat# 9102, RRID:AB_330744
phospho-ERK	Cell Signaling Technology	Cat# 9101, RRID:AB_331646
GFP	Santa Cruz Biotechnology	Cat# sc-9996, RRID:AB_627695
AKT	Cell Signaling Technology	Cat# 2920, RRID:AB_1147620
phospho-AKT (Thr308)	Cell Signaling Technology	Cat# 9275, RRID:AB_329828
phospho-AKT (Ser473)	Cell Signaling Technology	Cat# 9271, RRID:AB_329825
ubiquitin	Santa Cruz Biotechnology	Cat# sc-8017, RRID:AB_628423
HA tag	Abcam	Cat# ab16918, RRID:AB_302562
Caveolin-1	Cell Signaling Technology	Cat# 3267 RRID: AB_2275453
β-actin	Abcam	Cat# ab8226, RRID:AB_306371
CBL	Santa Cruz Biotechnology	Cat# sc-170, RRID:AB_2259627
transferrin receptor	Thermo Fisher Scientific	Cat# 13-6800, RRID:AB_2533029
Biological Samples		

Human lung samples	US Biomax	LC10012
Human lung samples	US Biomax	LC10013
Human lung samples and brain metastasis samples	China Medical University Hospital tissue biobank, Taichung, Taiwan	NA
Chemicals, Peptides, and Recombinant Proteins		
EGF	Sigma-Aldrich	Cat# E9644
Arsenic trioxide	TTY Biopharm Company	NA
OptiPrep density gradient medium	Alere Technologies AS, Oslo, Norway	Cat# AXS-1114542
IPTG	Sigma-Aldrich	Cat# I6758
Cycloheximide	Sigma-Aldrich	Cat# C7698
Chloroquine diphosphate salt	Sigma-Aldrich	Cat# C6628
MG132	Sigma-Aldrich	Cat# C2211
Erlotinib	Sigma-Aldrich	Cat# SML2156
Afatinib (*in vitro*)	Selleckchem	Cat# S1011
Afatinib (*in vivo*)	Abmole	Cat# M5307
Osimertinib (AZD9291)	MedChemExpress	Cat# HY-15772
Cetuximab	Merck	
Critical Commercial Assays		
fluo-4 (Fluo-4 NW Assay Kit;)	Thermo Fisher Scientific	Cat# F36205
Duolink In Situ Red Starter Kit Mouse/Rabbit (Immunofluorescence)	Sigma-Aldrich	Cat# DUO92101
Human RTK Phosphorylation Antibody Array -Membrane	Abcam	Cat# ab193662
Recombinant DNA		
BASP1 plasmid RC201815	OriGene	CAT#: RC201815
Lentiviral shRNA targeting BASP1 (clone E2)	National RNAi Core Facility, Taiwan	clone E2: TRCN0000281253
Lentiviral shRNA targeting BASP1(clone H1)	National RNAi Core Facility, Taiwan	clone H1: TRCN0000149347
Lentiviral shRNA targeting C-CBL (clone TRCN0000039727)	National RNAi Core Facility, Taiwan	clone TRCN0000039727

pLAS1w.3xLacO vector	National RNAi Core Facility, Taiwan	Cat# C6-7-18

Supplementary References

1. Lin CY, Chen HJ, Huang CC, Lai LC, Lu TP, Tseng GC, et al. ADAM9 promotes lung cancer metastases to brain by a plasminogen activator-based pathway. Cancer Res. 2014; 74: 5229-43.
2. Franken NA, Rodermond HM, Stap J, Haveman J, van Bree C. Clonogenic assay of cells in vitro. Nat Protoc. 2006; 1: 2315-9.
3. Huang TH, Huo L, Wang YN, Xia W, Wei Y, Chang SS, et al. Epidermal growth factor receptor potentiates MCM7-mediated DNA replication through tyrosine phosphorylation of Lyn kinase in human cancers. Cancer cell. 2013; 23: 796-810.
4. Taghibiglou C, Bradley CA, Gaertner T, Li Y, Wang Y, Wang YT. Mechanisms involved in cholesterol-induced neuronal insulin resistance. Neuropharmacology. 2009; 57: 268-76.
5. Chen PK, Chang BI, Kuo CH, Chen PS, Cho CF, Chang CF, et al. Thrombomodulin functions as a plasminogen receptor to modulate angiogenesis. FASEB J. 2013; 27: 4520-31.
6. Ma Y, Croxton R, Moorer RL, Cress WD. Identification of novel E2F1-regulated genes by microarray. Arch Biochem Biophys. 2002; 399: 212-24.
7. Amit I, Citri A, Shay T, Lu Y, Katz M, Zhang F, et al. A module of negative feedback regulators defines growth factor signaling. Nat Genet. 2007; 39: 503.

Supplementary Figures

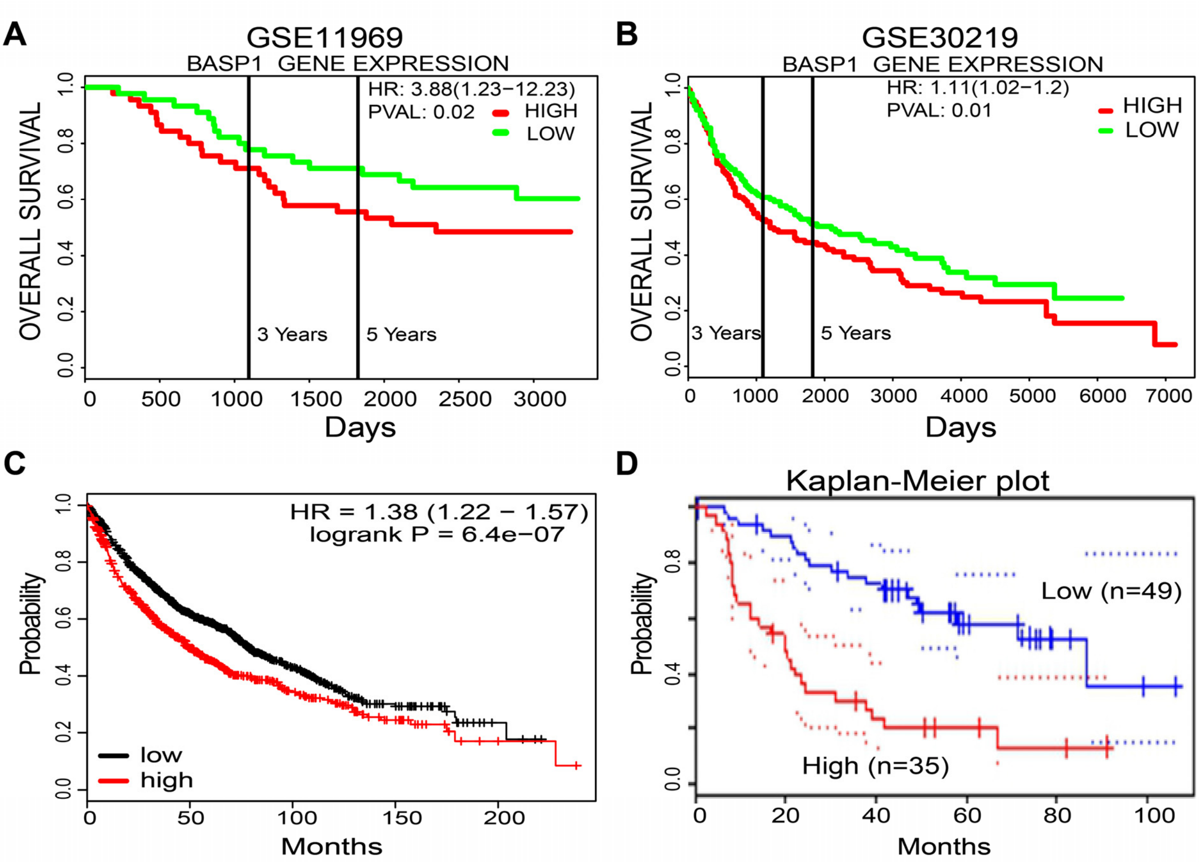

Figure S1, related to Figure 1. **(A-D)** Kaplan-Meier survival curves stratified by BASP1 expression from human lung cancer patient cohorts from GSE11969 (A), GSE30219 (B), Kaplan-Meier Plotter (C), and PrognoScan (D). The median of BASP1 expression was used as cutoff for low and high expression.

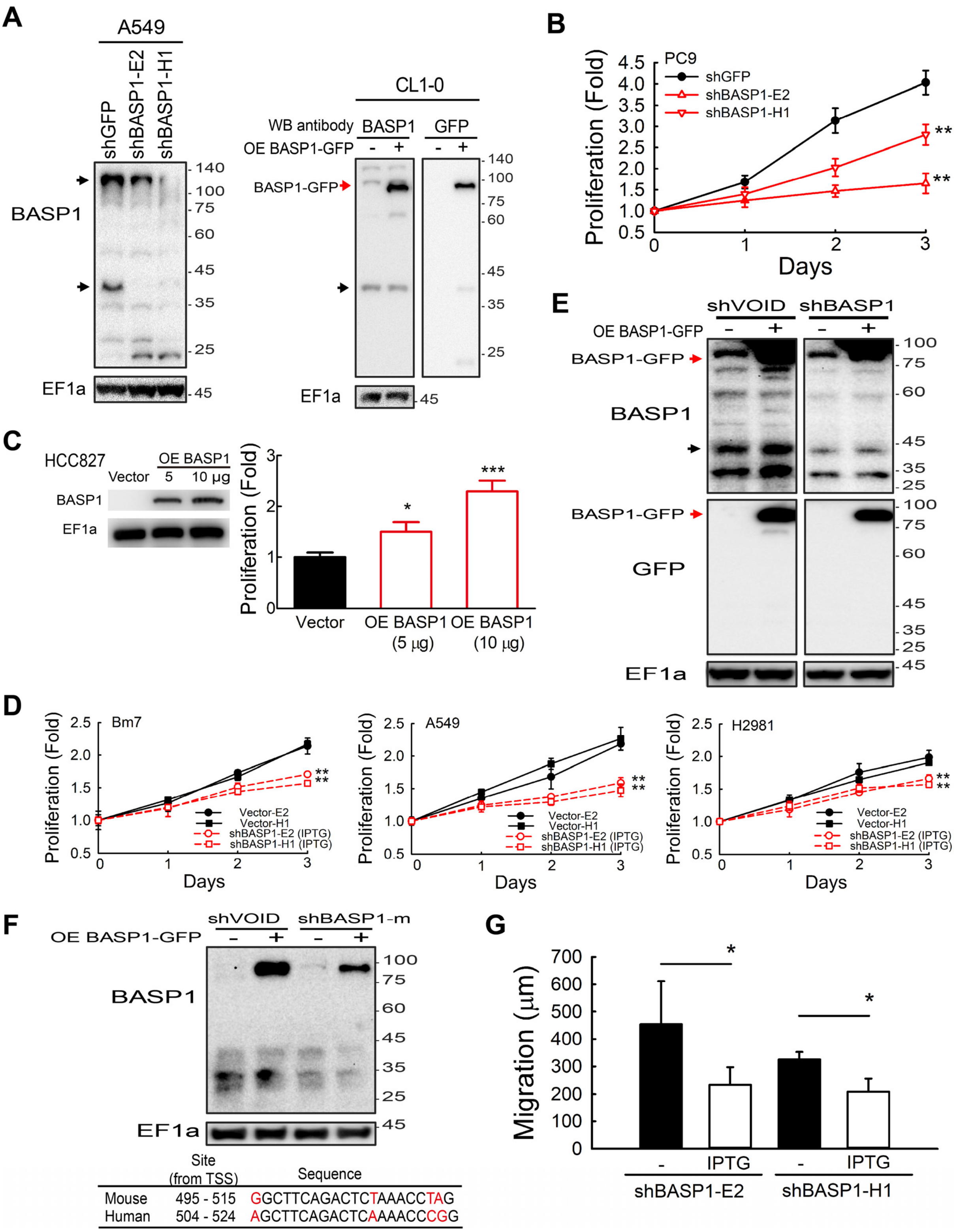

Figure S2, related to Figure 2. *BASP1*-knockdown influences cell proliferation and migration in lung cancer cells. **(A)** Western blot analysis of BASP1 in *BASP1*-knockdown A549 lung cancer cells (left). Endogenous BASP1 proteins and ectopic BASP1-GFP fusion proteins in CL1-0 cancer cells with overexpression (OE) of

BASP1-GFP fusion proteins were detected by BASP1 or GFP antibodies. Black arrow, endogenous BASP1; red arrow, BASP1-GFP. **(B)** The relative proliferation rate of control (shGFP) and *BASP1*-knockdown cells (shBASP1-E2 and shBASP1-H1) in PC9 cells by MTT assay. **(C)** Analysis of cell growth of HCC827 cells transfected with plasmids of BASP1 (OE BASP1) or vector alone. The amount of transfected plasmids is shown above the blots. **(D)** Cell proliferation of Bm7, A549, and H2981 in IPTG-induced BASP1 knockdown by MTT assays. **(E)** Western blot of BASP1 in control and *BASP1*-knockdown CL1-0 cells with overexpression of BASP1-GFP. **(F)** Western blot of control and *BASP1*-knockdown (shBASP1-mA) TC1 mouse lung cancer cells with OE of BASP1-GFP. The sequence of human BASP1 from BASP1-GFP plasmid and mouse BASP1 that was targeted by shRNA (shBASP1-m) was shown (bottom). The four different nucleotide sequences between human and mouse BASP1 genes are marked in red. **(G)** The 16-h migration distance of control and IPTG-inducible BASP1-knockdown Bm7 cells was measured by time-lapse video microscopy. At least 30 cells per experiment were recorded. Data represent mean ±SEM. A two-tailed Student's *t*-test was performed to compare control and treatment groups. *$P < 0.05$.

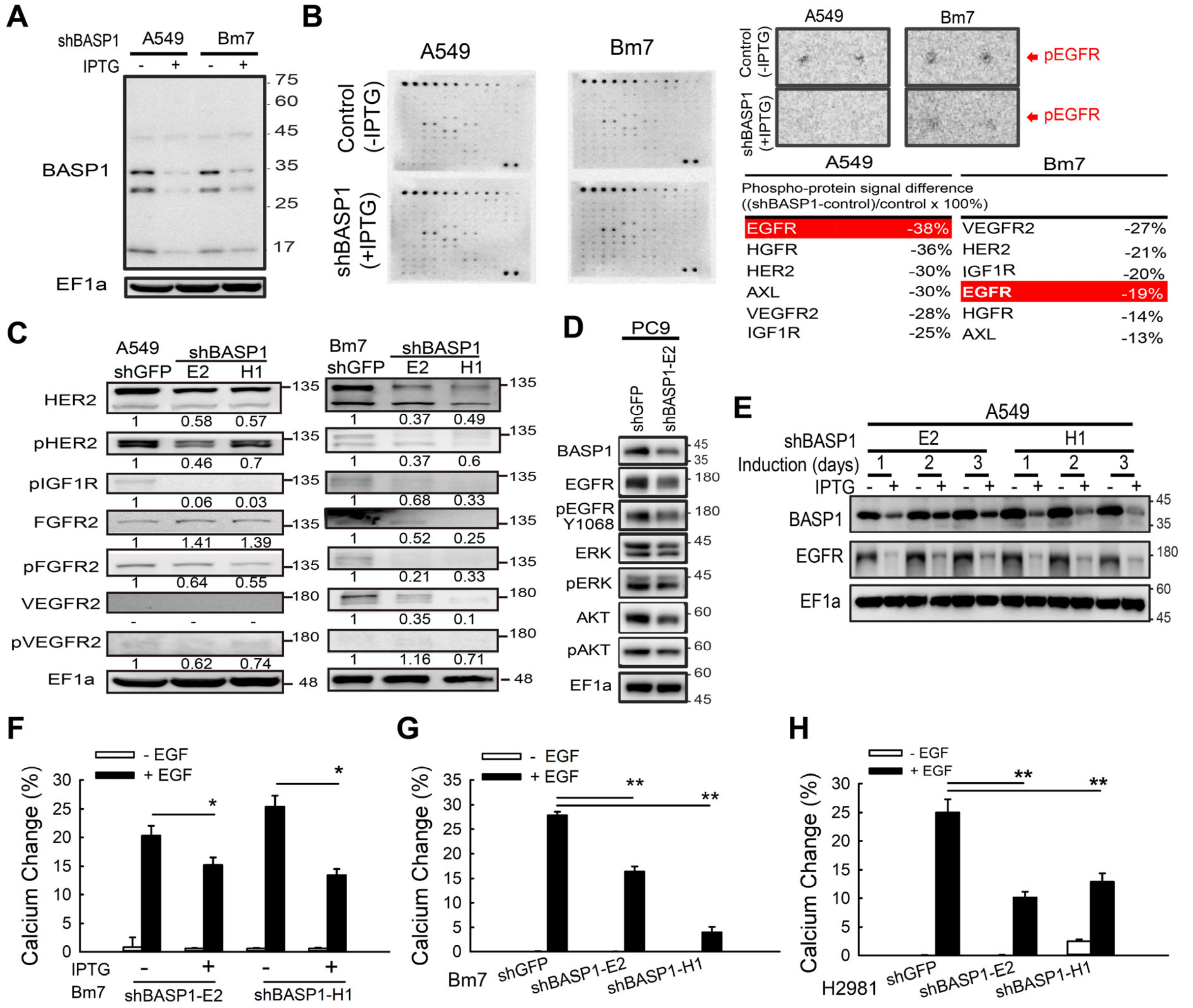

Figure S3, related to Figure 3. BASP1 increases EGFR protein expression in lung cancer cells. **(A)** Western blot of BASP1 in control and *BASP1*-knockdown lung cancer cells with IPTG-inducible shBASP1. **(B)** Cell lysates from control and BASP1 knockdown cells with IPTG-inducible shBASP1 were hybridized with human phospho-receptor tyrosine kinase (RTK) protein array. The differences in phospho-protein levels between control (−IPTG) and BASP1 knockdown (+IPTG) cells are shown on the right. **(C)** Western blot analysis of RTK and phospho-RTK in stable control and *BASP1*-knockdown A549 and Bm7 cells with the indicated antibodies. **(D)** Western blot of EGFR signaling pathways in PC9 lung cancer cells with IPTG inducible shBASP1 expression. **(E)** Western blot analysis of BASP1 in A549 lung cancer cells with IPTG-inducible shBASP1 at different days of IPTG induction with the indicated antibodies. **(F)** Intracellular calcium concentrations of control and IPTG-inducible *BASP1*-knockdown Bm7 lung cancer cells. Cells were serum starved for 4 hours and then treated with 50 ng/mL of EGF. **(G and H)** Intracellular calcium concentrations of stable control and *BASP1*-knockdown Bm7 (G) and H2981 (H)

lung cancer cells. All cells were serum starved for 16 hours followed by EGF (50 ng/mL) treatment.

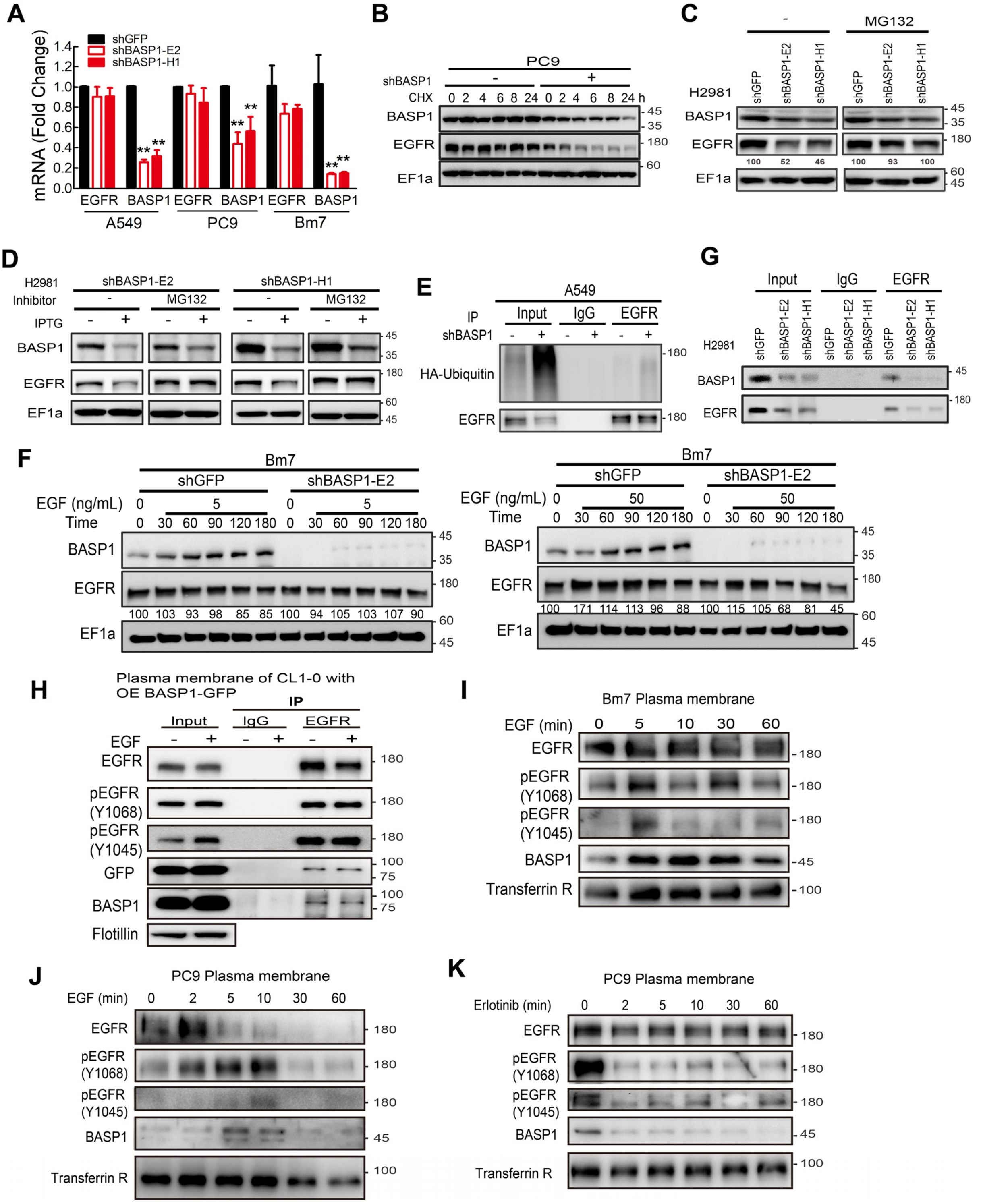

Figure S4, related to Figure 4 and Figure 5.

(A) Quantitative RT-PCR analysis of EGFR and BASP1 mRNA levels in control and *BASP1*-knockdown of A549, PC9, and Bm7 lung cancer cells. Data represent mean ± SD (n = 3). **(B)** Western blot analysis of BASP1 and EGFR in control and *BASP1*-knockdown PC9 lung cancer cells treated with CHX for indicated time periods. **(C)**

Western blot analysis of Control and *BASP1*-knockdown H2981 lung cancer cells cultured in starvation for 16 hours and then treated with EGF for 2 hours before collecting cell lysates. Cells were treated with 5 μM proteasome inhibitor MG132 for 3 hours before EGF stimulation. **(D)** Western blot of IPTG-induced *BASP1*-knockdown H2981 lung cancer cells cultured in starvation for 16 hours and then treated with EGF (50 ng/mL) for 2 hours before collecting cell lysate. Cells were treated with proteasome inhibitor MG132 (5 μM) for 3 hours before EGF stimulation. **(E)** IPTG-induced shBASP1 of A549 cells with HA-ubiquitin overexpression followed by MG132 and EGF treatment for 2 hours. EGFR was immunoprecipitated from cell extracts using an EGFR antibody. **(F)** Western blot analysis of EGFR of control and *BASP1*-knockdown Bm7 cells treated with low dose of EGF (5 ng/mL) or high dose of EGF (50 ng/mL) at indicated time points. **(G)** Coimmunoprecipitation of BASP1 and EGFR in H2981 lung cancer cells. EGFR was immunoprecipitated from the lysates followed by immunoblotting with the indicated antibodies. **(H)** Coimmunoprecipitation of EGFR and BASP1 from plasma membrane fraction of CL1-0 cells transiently transfected with BASP1-GFP. **(I** and **J)** Western blot analysis of plasma membrane fraction of Bm7 cells (I) and PC9 (J) treated with EGF (50 ng/mL) at indicated time points. **(K)** Western blot analysis of plasma membrane fraction of PC9 cells treated with Erlotinib (100 nM) and subsequently adding EGF for 5 min.

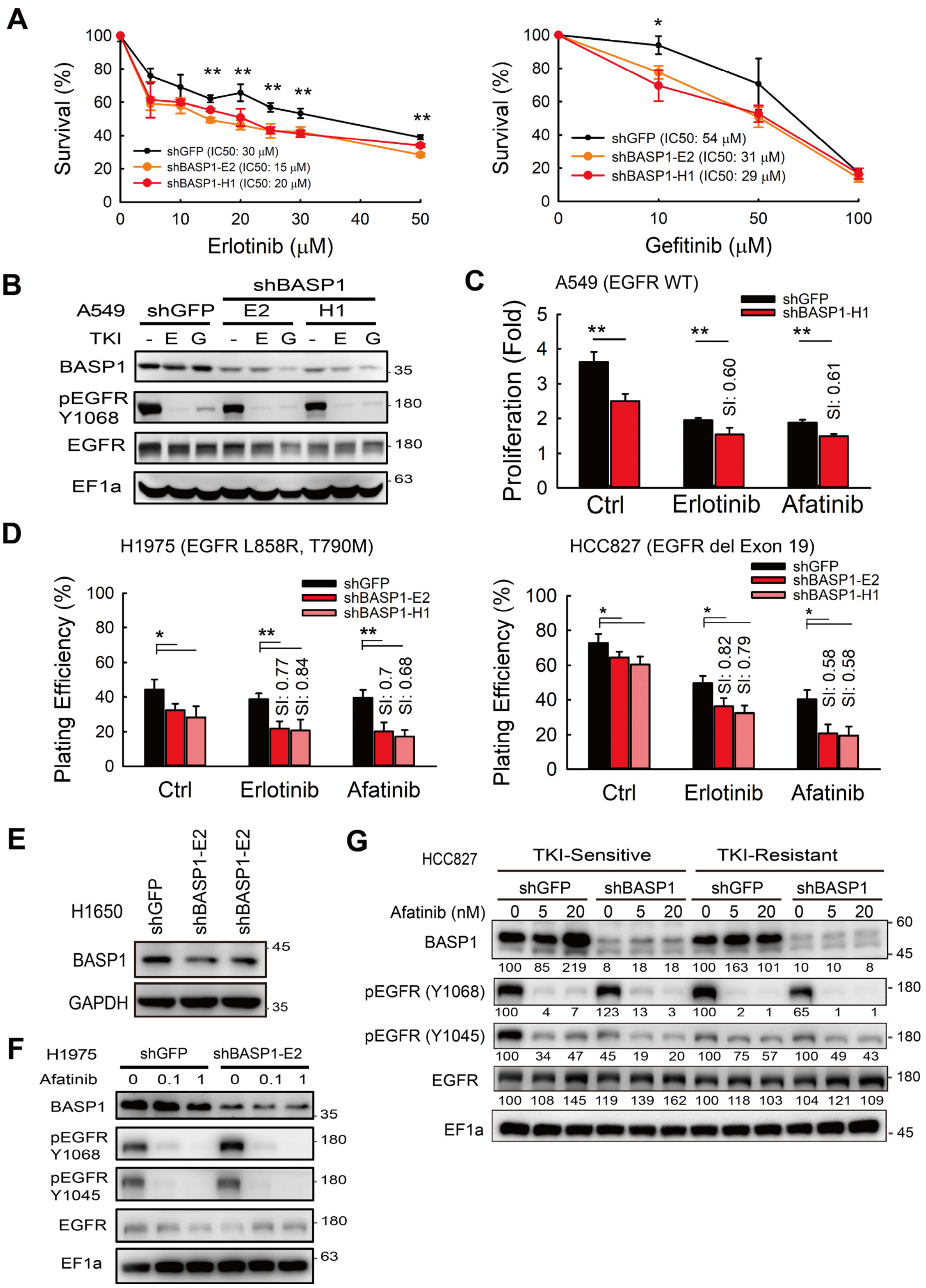

Figure S5, related to Figure 6.

(A) Cell proliferation of A549 cells treated with erlotinib and gefitinib for 2 days by MTT assay. **(B)** Western blot analysis of BASP1 and EGFR in control and *BASP1*-knockdown A549 lung cancer cells treated with erlotinib and gefitinib. E: erlotinib; G: gefitinib. **(C)** Comparison of cell proliferation of control or *BASP1*-knockdown of A549 treated with EGFR targeting agents, erlotinib and afatinib, by MTT assay. Erlotinib, 15 μM. Afatinib, 1 μM. **(D)** Plating efficiency of stable control and *BASP1*-knockdown H1975 and HCC827 lung cancer cells treated with EGFR TKIs by colony formation assay. Erlotinib (5 μM) and afatinib (0.1 μM) in H1975. Erlotinib (100 nM) and Afatinib (2.5 nM) in HCC827. **(E)** Western blot analysis of BASP1 in H1650 lung cancer cells. **(F)** Western blot analysis of BASP1 and EGFR in control and *BASP1*-knockdown H1975 lung cancer cells treated with afatinib. **(G)** TKI-Sensitive and TKI-Resistant HCC827 lung cancer cells were treated with afatinib for 1 h followed by EGF for 20 min. EGF, 50 ng/mL.

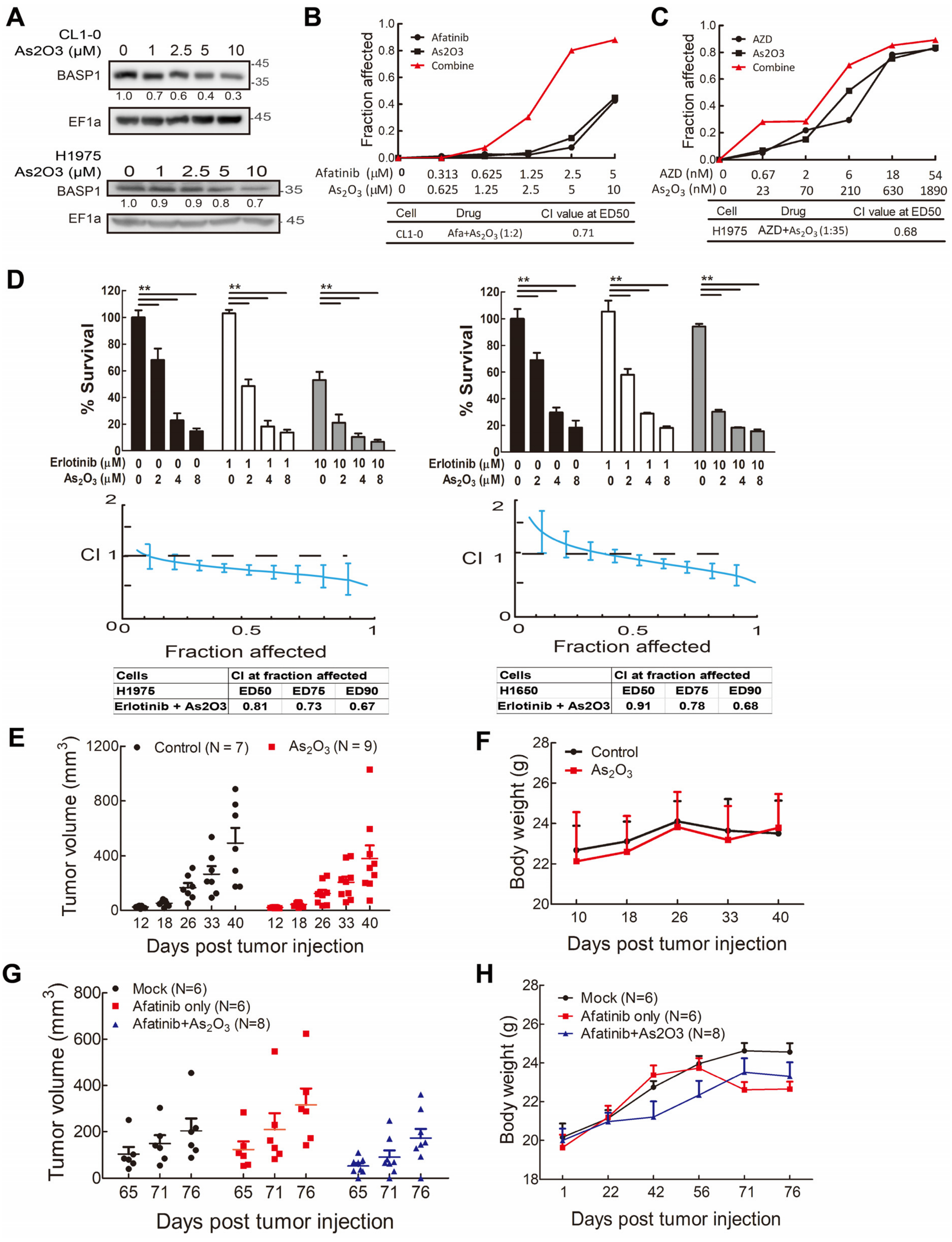

Figure S6, related to Figure 6.

(A) Western blot analysis of BASP1 in CL1-0, and H1975 lung cancer cells treated with arsenic trioxide. **(B)** Synergistic therapeutic effects of As_2O_3 and afatinib in CL1-0 cells by MTT assay. Combination index (CI) analysis (bottom). **(C)** Synergistic

therapeutic effects of As_2O_3 and AZD9291 in H1975 by colony formation assay. **(D)** H1975 and H1650 cells treated with As_2O_3 and erlotinib were subjected to MTT assay (top) and combination index (CI) curve analysis (bottom). CI < 1 indicates synergistic effect. **(E)** Tumor volume of SCID mice bearing subcutaneous H1975 lung cancer cells treated with As_2O_3**. (F)** Mouse body weight of SCID mice bearing subcutaneous H1975 lung cancer cells treated with As_2O_3. **(G)** Measured tumor volume from SCID mice bearing subcutaneous H1975 lung cancer cells treated with afatinib and As_2O_3**. (H)** Mouse body weight assessment from SCID mice bearing subcutaneous H1975 lung cancer cells treated with afatinib and As_2O_3.
